# Unveiling the Corrosion Mechanism of the Ni_*x*_Se_*y*_ Intermetallic Compound in Water-Based Solution

**DOI:** 10.1021/acsami.4c13794

**Published:** 2024-11-29

**Authors:** Jui-Teng Liang, Hwai-En Lin

**Affiliations:** † Institute of Mechatronic Engineering, National Taipei University of Technology, Taipei 10608, Taiwan; ‡ Graduate Institute of Manufacturing Technology, National Taipei University of Technology, Taipei 10608, Taiwan; § Department of Mechanical Engineering, National Taipei University of Technology, Taipei 10608, Taiwan

**Keywords:** NiSe, NiSe_2_, electrodeposition, intermetallic compound, corrosion

## Abstract

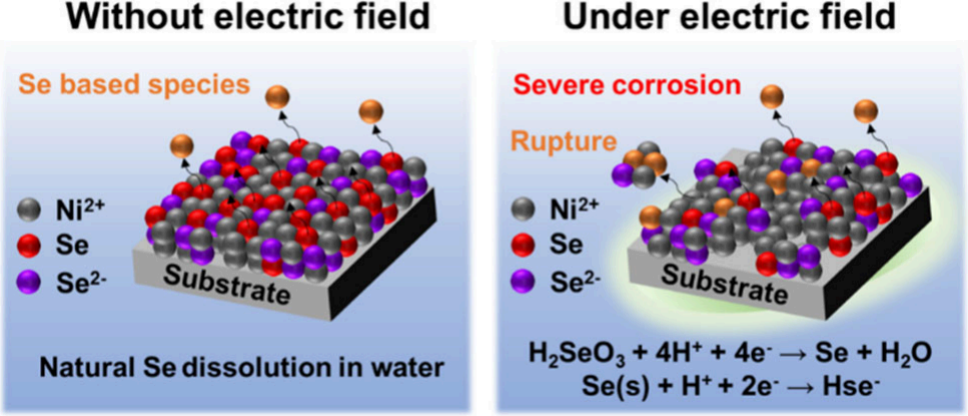

In this study, Ni–Se intermetallic compound coatings were fabricated using electrodeposition at various process temperatures (40–70 °C). The results show that increasing the process temperature promotes the deposition of Se, which leads to a transition in the crystal phase of the samples from the Ni_0.85_Se phase, prepared under low-temperature conditions, to a NiSe_2_ phase. The dissolution rate of Se in pure water from Ni–Se coatings is inversely related to the incorporated Se content, which indicates that the Ni_0.85_Se phase has a higher natural corrosion rate compared to the NiSe_2_ phase. Under the influence of an electric field, the corrosion behavior of Ni–Se coatings is dominated by a two-stage reaction involving Se transformation and dissolution. Coatings with a predominant Ni_0.85_Se phase exhibit more severe corrosion behavior compared to those with a predominant NiSe_2_ phase, which suggests that the corrosion reaction is enhanced by the electric field. However, because coatings with a predominant NiSe_2_ phase contain a higher proportion of Se_2_^2–^ ions, the dissolution reaction of SeO_*x*_, generated during the electrochemical reaction, is retarded, thereby inhibiting the progression of the corrosion reaction. Consequently, coatings with a predominant NiSe_2_ phase exhibit relatively better corrosion resistance in a water-based electrolyte.

## 1 Introduction

Ni–Se intermetallic compounds have garnered significant attention in recent years due to their unique properties, including the similar electronegativity of Ni and Se (i.e., χ_Ni_ = 1.9 and χ_Se_ = 2.4) and the distinctive valence electron configuration of nickel (3d^8^4s^2^).^1,2^ These factors enable Ni–Se intermetallic compounds to form a variety of compound compositions and microstructural morphologies, which offer considerable flexibility in material design for various applications. At ambient temperature, Ni–Se intermetallic compounds mainly exhibit three thermodynamically stable phases, which are Ni_1–*x*_Se (*x* = 0.85–1.00), NiSe_2_, and Ni_3_Se_2_. Ni–Se intermetallic compounds possess excellent electronic conductivity and electrochemical activity, which make them highly promising in the field of electrochemical energy storage, especially for applications as active materials in electrochemical catalysts and supercapacitor electrodes.^3,4^ In the realm of electrochemical catalysis, studies often compare the performance of Ni–Se intermetallic compounds to early research on transition metal oxides (TMOs). The chalcogens in transition metal chalcogenides (TM–X, TM = Fe, Co, Ni, etc.; X = S, Se, Te, etc.) have lower electronegativity than oxygen, which results in TM–X bonds with higher covalency degree and narrower band gaps. Moreover, the higher valence band edge of Ni–Se intermetallic compounds enables more efficient water oxidation reactions.^5^ Therefore, with optimized process parameters, Ni–Se-based electrodes can achieve better electrochemical performance compared to TMO materials. A major advantage of Ni–Se intermetallic compounds as electrochemical catalyst electrodes is their full water-splitting capability, which exhibits both oxygen evolution reaction (OER) and hydrogen evolution reaction (HER) characteristics in alkaline electrolytes.^6^ The inherent valence properties of Ni and Se allow both elements to act as hydrogen proton acceptors during water splitting, and the suitable hydrogen physisorption/chemisorption free energy of Ni–Se compounds effectively promotes the HER process.^7^ For the OER, the repulsion between Ni and Se in the 3d–2p orbitals, the multivalent characteristics of Se (which can be negatively charged), and the potential formation of NiOOH byproducts have all been demonstrated to positively affect the reaction.^8,9^ These advantages allow symmetrical electrode designs to efficiently perform both hydrogen and oxygen generation in a single system, which maximize device efficiency. On the other hand, Ni–Se intermetallic compounds show significant potential for supercapacitor applications. Their quasi-metallic electronic conductivity at room temperature, such as the presence of Ni–Ni bonds in Ni_3_Se_2_ crystals and the intrinsic paramagnetic metallic properties of NiSe_2_ (resistivity of >10^–3^ Ω cm),^10^ can enhance charge transfer reactions within the active material. This results in superior power and energy density benchmarks.^11^ In water-based supercapacitor configuration, the electrochemical reactions of Ni–Se intermetallic compounds often involve interactions with hydroxide ions in the electrolyte. This includes transformations such as NiSe/NiOOH (2NiSe + O_2_ + OH^–^ ↔ NiOOH + Se + e^–^) and Ni_3_Se_2_/NiOOH (2Ni_3_Se_2_ + 3O_2_ + 6OH^–^ ↔ 6NiOOH + 4Se + 6e^–^).^12,13^ These compounds exhibit excellent mechanical properties akin to metals and battery-type faradaic behavior, which enable them to maintain high power and energy density even when fabricated as flexible electrode.^14^

Ni–Se compounds are theoretically an excellent choice for the development of electrochemical catalysts, photoelectrochemical catalysts, and supercapacitor devices. However, in practical applications, using Ni–Se compounds as electrode materials in electrochemical systems faces significant corrosion issues. This problem arises mainly from the Se within the material, which easily forms highly water-soluble oxides/hydroxides. Without a thorough understanding of the corrosion reaction mechanisms and characteristics of Ni–Se compounds in water-based electrolytes, and without corresponding material design improvements, the durability limitations of these materials will greatly affect their practical application value. According to the literature review, it was found that there is no systematic investigation into this critical issue. This gap led to the inception of our study, which aims to determine whether Ni–Se compounds with different crystal structures exhibit varying corrosion reaction characteristics and whether the presence of an electric field exacerbates the corrosion reactions, which thereby affect electrode performance. In addition, a comparative analysis of the physical and chemical synthesis methods for Ni–Se compound coatings revealed that electrodeposition can produce uniform coatings in a single step.^15−17^ Furthermore, this method does not require the addition of binders or conductive carbon during electrode fabrication, which significantly enhances material utilization. Therefore, our team selected electrodeposition as the manufacturing process for Ni–Se electrodes. Notably, during the initial stages of our research, we observed that process temperature is a crucial factor influencing the crystal phase of electrodeposited Ni–Se coatings. We further controlled the crystal phase by varying the process temperature as an experimental variable and investigated the corresponding properties. This approach allowed us to elucidate the relationship between the crystal structures of Ni–Se intermetallic compounds and their material and electrochemical properties. In summary, the purpose of this study is to understand the corrosion characteristics of electrodeposited Ni–Se intermetallic compounds in water-based electrolyte systems. The data obtained could serve as important reference information for future development of Ni–Se coatings and the improvement of electrochemical electrode performance.

## 2 Experimental Details

### 2.1 Electrodeposition of Ni–Se Coating

The source materials for the electrodeposition of Ni–Se coatings were nickel sulfate hexahydrate (NiSO_4_·6H_2_O, 98%, Sigma-Aldrich) and selenium dioxide (SeO_2_, 99%, Emperor Chemical Co., Ltd.), with a molarity ratio of 1:1 (0.09 M) dissolved in deionized (DI) water. Additionally, 0.35 M sodium citrate dihydrate (Na_3_C_6_H_5_O_7_·2H_2_O, 99.5%, Thermo Fisher Scientific, Inc.) was added to the electrolyte to provide citrate ions during the electrodeposition process, which can react with Ni ions to form nickel citrate complexes and subsequently reduce to form the Ni coating. Ammonium chloride (NH_4_Cl, 99.8%, Scharlau) at 0.67 M and boric acid (H_3_BO_3_, 100%, Honeywell Fluka, Ltd.) at 0.32 M were added as the conductive salt and buffer, respectively. After preparing the electrolyte, ammonia solution (NH_4_OH, 25%, Honeywell Fluka, Ltd.) was added to adjust the solution to pH 7, then completing the electrolyte preparation.

A SUS420 stainless-steel sheet (Key Star Electron Co., Ltd.) with a diameter of 12.5 mm and thickness of 0.8 mm was selected as the substrate. Prior to electrodeposition, the substrate was polished sequentially with SiC sandpapers of grit numbers 180, 400, 800, 1200, and 2000 and then placed in a vacuum oven to remove moisture. The dried substrate was then immersed in 2 wt % sodium hydroxide (NaOH, 97%, Thermo Fisher Scientific, Inc.) for surface degreasing, rinsed with DI water, and dipped in hydrochloric acid solution (HCl, 36.5–38.0%, Honeywell Fluka, Ltd.) for 10 s to remove surface oxides, finally followed by ten rinses (each for at least 30 s) with DI water to complete the pretreatment steps. The electrodeposition process employed a two-electrode system, with the pretreated SUS420 stainless-steel substrate as the working electrode (cathode) and a titanium rod as the inert anode. The electrodeposition was carried out at a current density of 100 mA cm^–2^ for 10 min. Given the relatively low current density and short electrodeposition time, the changes in electrolyte concentration due to the use of an inert anode were considered negligible. Initial experimental results indicated that the bath temperature is the main factor that influences the crystal structure of the electrodeposited Ni–Se coating. To ensure a consistent deposition rate and avoid concentration changes due to electrolyte evaporation at high temperatures, the process temperature was controlled between 40 and 70 °C in increments of 10 °C. After deposition, the samples were dried in a vacuum oven at 60 °C. Subsequent material and electrochemical property tests were conducted to observe phase transitions and analyze the relationship between the crystalline phases of the coatings and their corresponding material and electrochemical properties. The samples prepared at four different bath temperatures were named NS-40, NS-50, NS-60, and NS-70, respectively.

### 2.2 Material Characterization

A field emission scanning electron microscope (FE-SEM, 8100 FE-SEM, HITACHI Regulus) was used to observe the surface morphology and cross-sectional characteristics of the electrodeposited Ni–Se coatings. Three repeat tests were conducted for the thickness measurements. Each measurement point was evaluated at the same magnification across five different locations, and the average values were used for quantitative comparison. Additionally, a three-dimensional optical microscope (VR-3100, KEYENCE) was employed to create three-dimensional (3D) surface morphology maps of the samples using optical imaging method. The measured area values (*A*) and corresponding two-dimensional coordinates [*Z*(*x*,*y*)] were substituted into the formula for calculating the arithmetic mean height (as shown in eq 1) to evaluate the surface roughness of the samples.
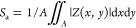
1To determine the chemical composition of the samples prepared with different temperatures, energy-dispersive spectroscopy (EDS, X-Max Extreme, Oxford Instruments plc) integrated with FE-SEM was used for elemental analysis of the coatings. The accelerating voltage was set to either 10 or 15 kV, depending upon the specific sample. The average elemental ratios of each coating were then applied to the modified Faraday’s law of electrolysis to calculate the cathodic current efficiency (η) of the electrodeposition process.^18^ The calculation formula is as follows:
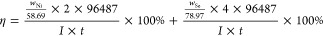
2where *w*_Ni_ and *w*_Se_ represent the mass of nickel and selenium, respectively, in the electrodeposited coating measured after drying. *I* denotes the operating current (*A*), and *t* is the deposition time (s). Besides, the EDS analysis of sample was carried out after the corrosion test as well to clarified the corrosion mechanism. The surface chemical states of the elements in the samples were analyzed using X-ray photoelectron spectroscopy (XPS, Thermo VG-Scientific/Sigma Probe, Thermo Fisher Scientific, Inc.) with an Al Kα (1486.6 eV) target. The measured spectra were first calibrated using the binding energy of the C–C bond in the spin–orbit C 1s (284.8 eV), and then the data were processed using XPSPEAK41 software with Lorentzian/Gaussian functions for background simulation and peak fitting. The crystal phases of the coatings were analyzed using grazing incidence X-ray diffraction (GI-XRD, D8 ADVANCE, Bruker) with a Cu Kα radiation source. The samples were scanned over a 2θ range of 20° to 80° with an incident angle below 5°. In addition to basic material properties, the hardness of the coatings was evaluated using a Vickers hardness tester (1202, Wilson Tukon). A diamond indenter with a pyramidal shape and an angle of 136.5° ± 0.5° was used, which applied a load of 0.01 N for 15 s to create an indentation on the coating surface. The hardness value was calculated using the formula^19^
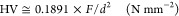
3where *F* is the applied load (N) and *d* is the average length of the indentation diagonal (mm). Each sample was tested at least 20 different points on the surface, and the average value and standard deviation were reported to ensure the validity of the results. To understand the natural dissolution characteristics of the samples in pure water, the electrodeposited Ni–Se coatings were immersed in DI water, and the dissolution of elements over time was assessed using inductively coupled plasma optical emission spectrometry (ICP–OES, Optima 8000, PerkinElmer).

### 2.3 Electrochemical Measurements

To understand the electrochemical behavior of Ni–Se coatings prepared at different bath temperatures in saline solution, cyclic voltammetry (CV), open circuit potential (OCP), potentiodynamic polarization (PDP), and electrochemical impedance spectroscopy (EIS) analyses were conducted using an electrochemical workstation (VersaSTAT 3F, AMETEK) at room temperature (20 ± 5 °C). The electrolyte used was a 3.5 wt % NaCl (99.5%, Honeywell Fluka, Ltd.) water-based solution, which simulated a high-salt environment. The experimental setup was a three-electrode system, which comprised the electrodeposited Ni–Se coating as the working electrode, a platinum counter electrode, and an Ag/AgCl|sat. KCl reference electrode. The exposed area of the working electrode was fixed at 1 cm^2^. The CV tests aimed to investigate the redox potentials and electrochemical behavior of the samples in saline solution, with a potential window set from 0.0 to −1.0 V versus Ag/AgCl|sat. KCl and a scan rate of 1 mV s^–1^. Prior to PDP testing, all samples underwent a 1 h OCP scan to stabilize the electrode surface potential. The PDP potential window was based on the stable potential obtained from the 1 h OCP test, set at ±0.30 and ±0.60 V versus Ag/AgCl|sat. KCl, with a scan rate of 1 mV s^–1^. After completing the PDP test, the polarization curves (±0.30 V versus Ag/AgCl|sat. KCl) were analyzed using the Tafel extrapolation method. A tangent line was drawn on the anodic polarization curve within the range of corrosion potential +50 mV, and the intersection of this tangent with a horizontal line at the corrosion potential was used to evaluate the corrosion current. EIS analysis was performed at 0, −0.64, and −0.74 versus Ag/AgCl|sat. KCl. The input AC signal was a sine wave with a peak amplitude of 10 mV, and the frequency range was set from 1 to 10 000 Hz. The impedance spectra were analyzed in two parts. First, the experimental data were presented as Nyquist plots and fitted using Zview software with various equivalent circuits. All calculated maximum χ^2^ values were below 0.5% to ensure the reliability of the fitting results. Second, the impedance data were processed using the relaxation time inverse distribution tool proposed by Wan et al.,^20^ with the results presented as a normalized relaxation time distribution function (γ, Ω cm^2^ s) versus frequency (Hz) for a more intuitive comparison with the Nyquist plots.^21^

## 3 Results and Discussion

### 3.1 Characteristics and Chemical Composition of Electrodeposited Ni–Se Coatings

**Figure 1:**
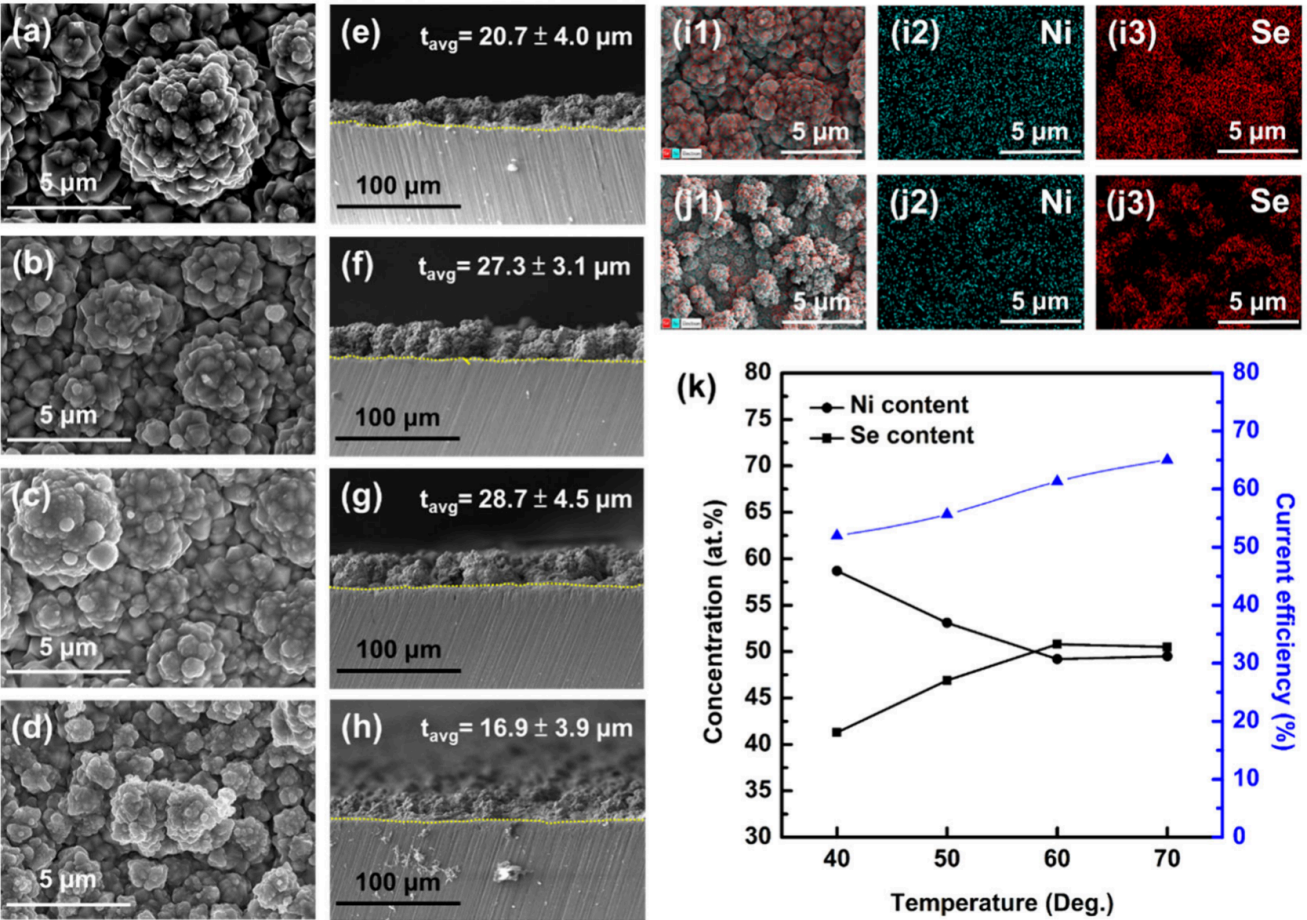
FE-SEM micrographs showing the surface morphology (secondary electron, SE mode) of (a) NS-40, (b) NS-50, (c) NS-60, and (d) NS-70 samples, along with cross-sectional images (backscattered electron, BSE mode) of (e) NS-40, (f) NS-50, (g) NS-60, and (h) NS-70 samples. The yellow dashed line indicates the interface between the SUS420 substrate and the Ni–Se coating. Panels i1 and j1 illustrate the surface morphology and overlaid element distribution of Ni and Se for the NS-50 and NS-70 samples, respectively. Panels i2, i3, j2, and j3 display EDS mapping results for Ni and Se elements for the NS-50 and NS-70 samples. Panel k compares the Ni/Se concentration in coatings prepared at different process temperatures and their corresponding cathodic current efficiency.

The surface morphologies of electrodeposited Ni–Se coatings prepared at different process temperatures are shown in panels a–d of Figure 1. It can be observed that the NS-40 and NS-50 samples are primarily composed of rhombohedral crystals with a cauliflower-like morphology, with sizes ranging from several hundred nanometers to a few micrometers. When the process temperature is increased to 60 °C, the underlying layer of the coating still maintains the rhombohedral crystal characteristics; however, the surface is covered with finer particles that transition to spherical agglomerates. Further increasing the process temperature to 70 °C results in more spherical agglomerates adhering to the surface. From these observations, three preliminary inference can be drawn: first, the process temperature would affect the material properties of Ni–Se coatings; second, the spherical agglomerates on the top layer and the rhombohedral stacking at the bottom may have different chemical compositions and/or crystalline characteristics; and third, the formation of Ni–Se compounds during electrodeposition may involve multiple stages. Panels e–h of Figure 1 show the cross-sectional characteristics of Ni–Se coatings prepared at different process temperatures, with an average coating thickness ranging from 16 to 29 μm. The highest and lowest average thicknesses were found in the NS-60 and the NS-70 samples, which measured 28.7 μm (max, 35.9 μm; min, 18.3 μm) and 16.9 μm (max, 23.8 μm; min, 10.7 μm), respectively. To clearly distinguish the interface between the coating and the substrate, FE-SEM EDS was used to perform elemental mapping on the cross-section of the sample, with the analysis results shown in Figure S1 of the Supporting Information. Given the total electrodeposition time of 10 min, the average deposition rate of Ni–Se coatings under the conditions of this study is approximately 1.6–2.9 μm min^–1^. Panels i1–i3 and j1–j3 of Figure 1 display the FE-SEM surface EDS mapping results for the NS-50 and NS-70 samples, respectively. Ni is uniformly distributed in both samples, whereas Se is predominantly located in the surface agglomerates. This observation supports the previous inference that the rhombohedral stacking in the bottom layer has a higher Ni content, whereas the surface agglomerates likely consist of compounds formed by Ni and Se. Further investigations using EDS point analysis to evaluate the chemical compositions of different samples and the corresponding cathodic current efficiency are shown in Figure 1k. The results indicate that the samples consist of Ni and Se. As the process temperature increases from 40 to 60 °C, the Ni content in the coatings decreases, while the Se content shows the opposite trend. When the bath temperature is further increased to 70 °C, the Ni content does not decrease further, while the Se content stabilizes. The cathodic current efficiency of Ni–Se compound electrodeposition, under the process parameters set in this study, ranges from 51 to 66%, which is lower than the typical value for pure Ni coating fabricated by electrodeposition (around 90%).^22^ Moreover, the variation in cathodic current efficiency is significantly related to changes in bath temperature. The primary influences of bath temperature are 2-fold: first, the amount of deposition increases with rising bath temperature (approximately from 9.1 to 11.3 mg); second, the calculated results show that increasing the bath temperature does not significantly affect the current efficiency of Ni deposition (approximately between 18 and 22%). However, it does increase the current efficiency of Se deposition by about 9.7% (from approximately 33.9 to 43.6%). Therefore, the observed increase in cathodic current efficiency is likely a comprehensive result of the increment in deposit amounts and the increased cathodic current efficiency of Se. To assess the uniformity of element distribution in the coating, we performed an EDS line scan analysis on the cross-section of the samples. The results, shown in Figure S2 of the Supporting Information, reveal that Ni and Se in the coating are evenly distributed, except for signals from the SUS420 substrate and the resin used to seal the samples. Interestingly, the Se content in the coating increases with the process temperature. At bath temperatures of 40 and 50 °C, the Ni content in the coating is higher than the Se content. However, when the bath temperature rises above 60 °C, the Se content exceeds the Ni content. This observation is consistent with the variation trend of current efficiency and chemical composition shown in Figure 1k, which suggests that changes in bath temperature not only alter the chemical composition of the coating but may also induce changes in the crystal phase.

### 3.2 Crystal Structure of Electrodeposited Ni–Se Coatings

**Figure 2:**
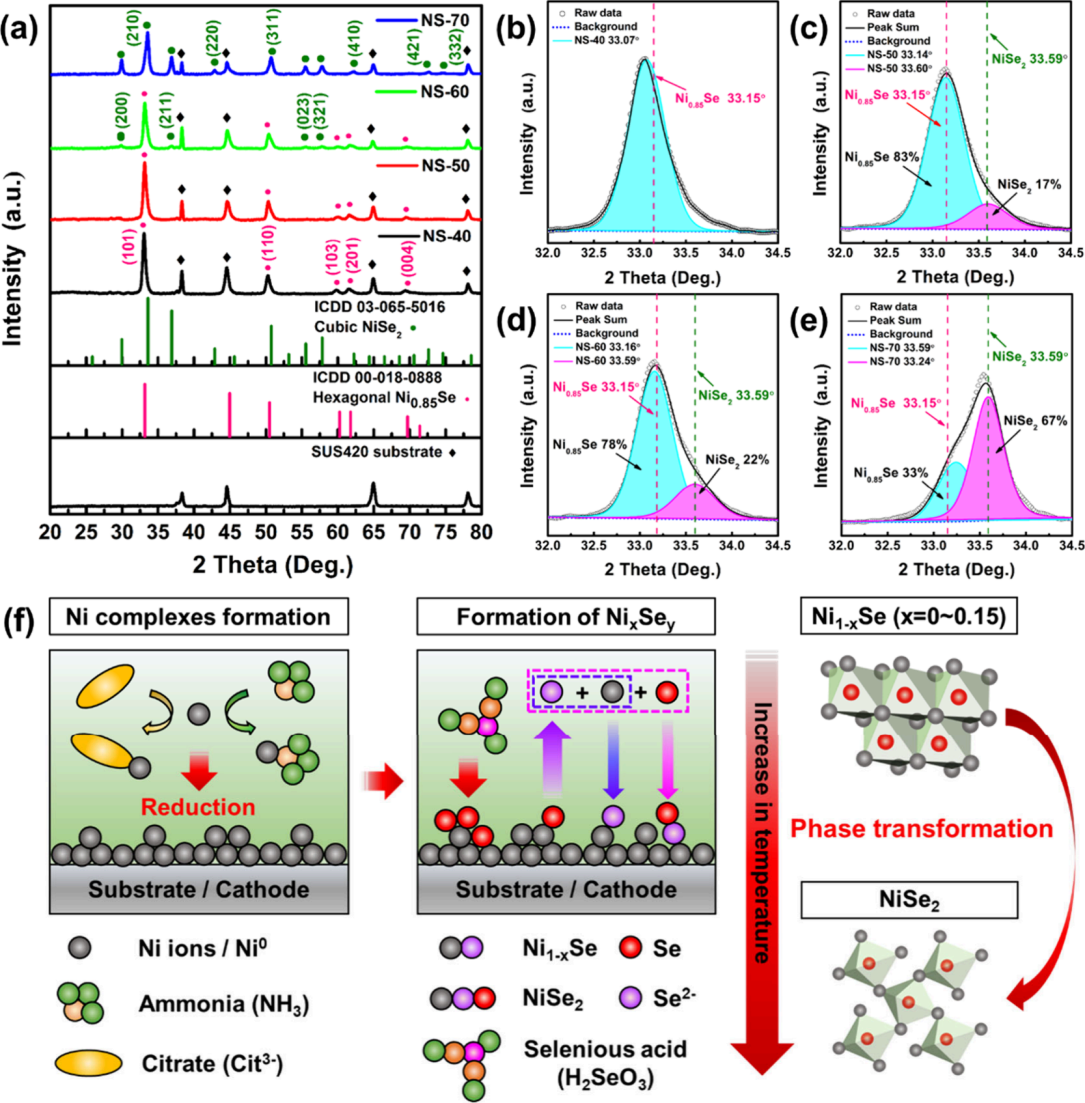
Panel a shows the XRD spectra of Ni–Se coatings prepared at different process temperatures. Panels b–e illustrate the shift of the preferential diffraction peak around 33° (2θ) and the phase transition of Ni–Se compounds with increasing process temperature. Panel f provides a schematic illustration of the possible film formation mechanism of Ni–Se compounds during the electrodeposition process.

From the previous discussion on the changes in surface morphology and chemical composition of Ni–Se coatings with varying process temperatures, it can be inferred that the crystalline characteristics of the coatings might also change. This inference can be confirmed by the XRD analysis shown in Figure 2a. Apart from the substrate signals, the crystalline features of the as-prepared Ni–Se coatings mainly consist of hexagonal Ni_0.85_Se (ICDD 00-018-0888) and cubic NiSe_2_ (ICDD 03-065-5016). Analyzing the diffraction peak positions, the NS-40 and NS-50 samples exhibit characteristics of nearly pure-phase Ni_0.85_Se. The preferred orientation is the (101) plane at 33.15°. When the process temperature is raised to 60 °C, a phase transition occurs in the electrodeposited Ni–Se coatings, and peaks corresponding to NiSe_2_ appear alongside those of Ni_0.85_Se. The diffraction peaks at 29.96°, 36.91°, 55.54°, and 57.83° correspond to the (200), (211), (023), and (321) planes of NiSe_2_. Further increasing the temperature to 70 °C results in a complete phase transition to NiSe_2_. The preferred orientation is the (210) plane at 33.59°.

To fully understand the reaction characteristics leading to the phase transition in electrodeposited Ni–Se coatings due to varying bath temperatures, the peak fitting of the preferred orientation of Ni_0.85_Se and NiSe_2_ were further conducted, as shown in panels b–e of Figure 2. Figure 2b shows that the main peak of the NS-40 sample exhibits characteristics of pure-phase Ni_0.85_Se, with the peak position (2θ = 33.07°) slightly shifted by 0.08° to a lower angle compared to the standard position (2θ = 33.15°) of the Ni_0.85_Se (101) plane, indicating a slight lattice expansion according to Bragg’s law:
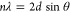
4When the process temperature is increased to 50 °C (as shown in Figure 2c), the electrodeposited Ni–Se coatings begin to show crystallographic features corresponding to NiSe_2_ (2θ = 33.60°). Additionally, the diffraction peak position of Ni_0.85_Se further shifts slightly to a higher angle (2θ = 33.14°) compared to the NS-40 sample, closer to the standard peak position. The calculated proportions of Ni_0.85_Se and NiSe_2_ are approximately 83 and 17%, respectively. As shown in Figure 2d, the proportion of NiSe_2_ increases further to 22% when the process temperature increases to 60 °C, with the diffraction peak position of Ni_0.85_Se also shifting to a higher angle (2θ = 33.16°). When the process temperature is raised to 70 °C, the main peak characteristics of the electrodeposited Ni–Se coatings are dominated by the NiSe_2_ phase (as shown in Figure 2e), with the proportions of Ni_0.85_Se and NiSe_2_ being approximately 33 and 67%, respectively. On the basis of the results showing in panels a and e of Figure 2, it can be inferred that the transition from rhombohedral to spherical grains observed in panels a–d of Figure 1 is related to the increased Se content in the coatings (as shown in Figure 1k) and the sufficient energy supply for phase transition of the Ni–Se compounds at higher process temperature.

Analysis reveals differences in the surface and base characteristics of electrodeposited Ni–Se coatings, an uneven distribution of Se elements in EDS mapping, and phase transitions with varying process temperatures. These observations suggest a preliminary formation mechanism during the electrodeposition process, as illustrated in Figure 2f. The deposition reactions of Ni–Se intermetallic compounds during the electrochemical procedure can be categorized into two types. In the first type of reaction, citrate (Cit^3–^) and ammonia (NH_3_) in the citric acid-ammonia electrolyte system would form complexes with Ni^2+^ ions, which result in the formation of [Ni(Cit)_n_]^m-^ and [(Ni(NH_3_)_n_]^2+^ ions. Due to the limited amount of Ni^2+^ ions in the solution, there is a competitive effect between citrate and ammonia. Under the influence of an electric field, these complexes would be reduced on the cathode surface to form a metallic Ni coating, as described by reactions 1 [Ni(Cit)]^−^ + 2e^–^ → Ni(s) + Cit^3–^ and 2 [(Ni(NH_3_)_6_]^2+^ + 2e^–^ → Ni(s) + 6NH_3_.^23^ On the other hand, SeO_2_ in the electrolyte undergoes hydrolysis to produce selenous acid (SeO_2_ + H_2_O → H_2_SeO_3_), which is reduced in the aqueous solution to form Se (H_2_SeO_3_ + 4H^+^ + 4e^–^ → Se(s) + 3H_2_O).^24^ Under the influence of an electric field, Se undergoes disproportionation to produce Se^2–^ and SeO_3_^2–^ ions (3Se + 6OH^–^ → 2Se^2–^ + SeO_3_^2–^ + 3H_2_O)^2^. The concentration of Se^2–^ ions redissolved in the electrolyte from the Se deposited on the cathode surface varies with the reaction energy provided at different process temperatures. At relatively low operating temperatures (i.e., 40–50 °C), most of the Se on the surface undergoes disproportionation to form Se^2–^ ions, which react with Ni^2+^ ions to form Ni_1–*x*_Se [i.e., (1 – *x*)Ni^2+^ + Se^2–^ → Ni_1–*x*_Se, where *x* = 0–0.15].^12^ When the process temperature rises to a certain level (i.e., 60–70 °C), the reduction of Se on the cathode surface is enhanced, which results in the formation of more Se. However, within the limited electrodeposition time, only a portion of the Se is reduced to Se^2–^ ions, with the remaining Se reacting with Se^2–^ ions to form Se_2_^2–^, which then reacts with Ni^2+^ ions to produce NiSe_2_ (i.e., Ni^2+^ + Se_2_^2–^ → NiSe_2_).^25^ In summary, in the early stages of the deposition reaction, the primary component of the coating is metallic Ni, which is directly reduced from [Ni(Cit)_*n*_]^*m*−^ and [(Ni(NH_3_)_*n*_]^2+^ complexes, as evidenced by the EDS mapping results of the base layer of the coating. Subsequently, Se gradually forms on the coating surface and undergoes disproportionation under the electric field to produce Se^2–^ ions. The amount of Se^2–^ ions generated varies with the process temperature, which leads to differences in the phase composition of Ni–Se compounds. It is speculated that the formation of the NiSe_2_ phase is related to Se that has not undergone disproportionation on the surface, which results in the uneven distribution of Se in the coating, as shown in panels j1–j3 of Figure 1.

**Figure 3:**
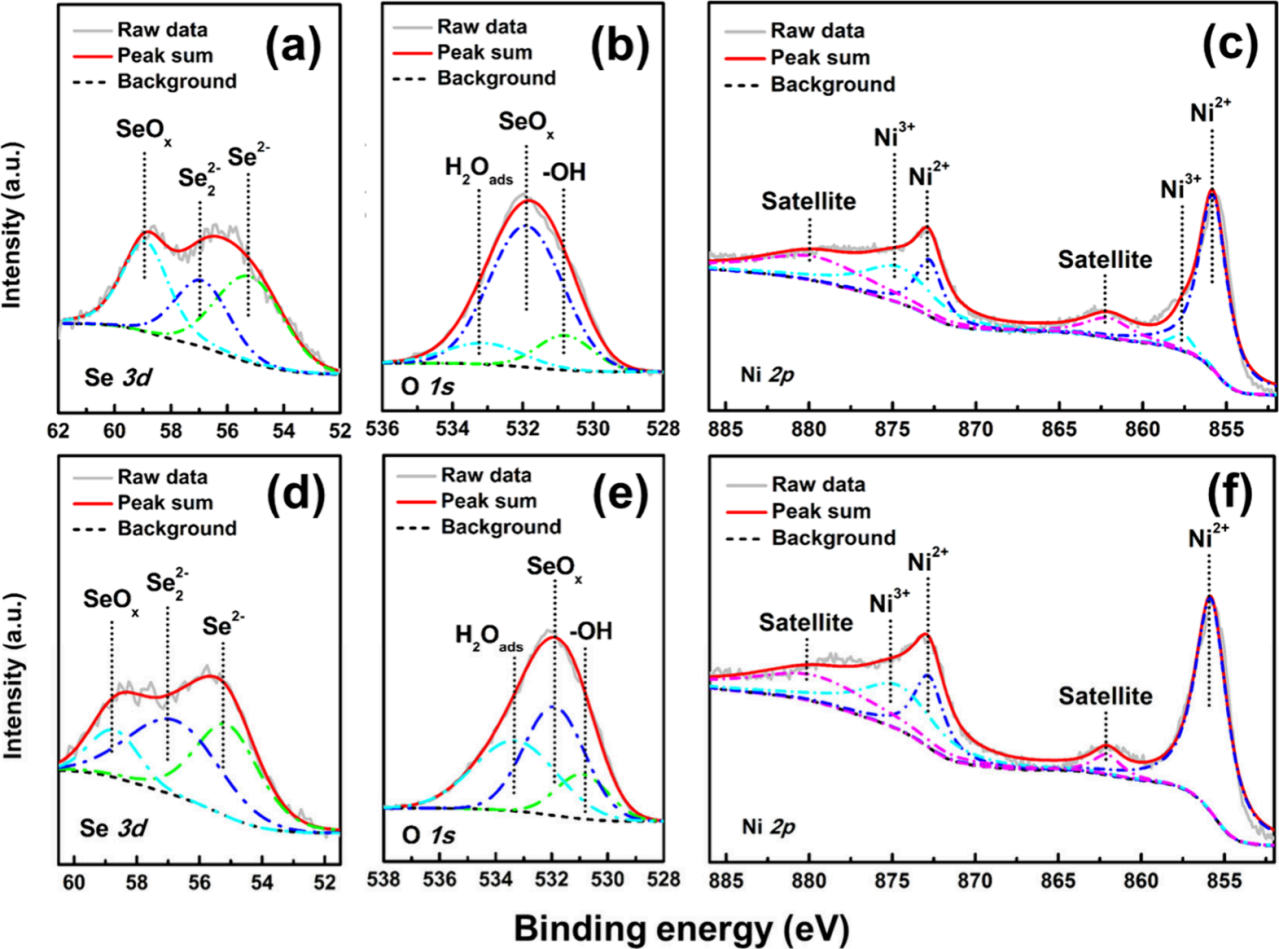
XPS spectra for (a) Se 3d, (b) O 1s, and (c) Ni 2p orbits of the NS-50 sample. Panels d–f are core-level Se 3d, O 1s, and Ni 2p regions of the NS-70 sample, respectively.

The chemical composition of the as-prepared Ni–Se coatings was further investigated using XPS analysis. From the XRD analysis results in Figure 2, it is evident that the crystal structures of the NS-50 and NS-70 samples are predominantly Ni_0.85_Se and NiSe_2_, respectively, and exhibit characteristics close to pure phases. Thus, these two samples were selected for subsequent analysis of the valence states of the coatings, and the results are shown in Figure S3 of the Supporting Information and Figure 3. Figure S3 of the Supporting Information indicates that, aside from C, O, Ni, and Se, no other impurity signal was detected in the samples. Both NS-50 and NS-70 samples show Se^2–^ and Se_2_^2–^ characteristics in the spin–orbit Se 3d_5/2_ region (panels a and d of Figure 3), with binding energies of 55.2 and 56.9 eV, respectively, which provide evidence for the formation of Ni–Se intermetallic compounds on the coating surface.^26^ Additionally, a peak at 58.8 eV corresponds to SeO_*x*_, associated with the natural oxidation of some surface Se elements in the air.^27^ Panels b and e of Figure 3 reveal that the O 1s orbital spectra of both NS-50 and NS-70 samples can be deconvoluted into three peaks representing hydroxyl groups (530.8–530.9 eV), surface oxides (e.g., SeO_*x*_, 531.9 eV), and surface physisorbed/chemisorbed H_2_O (533.1–533.3 eV).^28−30^ Comparing the relative intensities of these peaks, SeO_*x*_ species dominate the surface of both samples, with the main difference being that the H_2_O_ads_ peak intensity is higher in the NS-70 sample compared to the NS-50 sample. This, combined with the Ni_0.85_Se/NiSe_2_ phase analysis from panels c and e of Figure 2, suggests that the NiSe_2_ phase may have better surface water adsorption properties, potentially promoting natural oxidation/hydroxylation of the Ni–Se intermetallic compounds. According to panels c and f of Figure 3, the Ni 2p orbital spectra of the NS-50 and NS-70 samples show similar characteristics. The NS-50 sample can be deconvoluted into six peaks: Ni^2+^ (855.8 eV), and Ni^3+^ (857.6 eV) in the Ni 2p_3/2_ region, and Ni^2+^ (872.8 eV) and Ni^3+^ (874.6 eV) in the Ni 2p_1/2_ region.^31−34^ Additionally, broad bands at 862.1 and 879.6 eV correspond to the shakeup satellites of Ni 2p_3/2_ and Ni 2p_1/2_, respectively, which indicate the presence of Ni^2+^.^35^ For the NS-70 sample, the spectrum comprises Ni^2+^ (855.8 and 872.8 eV), Ni^3+^ (874.6 eV), and satellite peaks (862.1 and 879.6 eV). In summary, the Ni^2+^, and Ni^3+^ features within the coatings correspond to the Ni–Se bonding and the possible formation of surface oxides or hydroxides during the electrodeposition process. These results corroborate the XRD analysis results in Figure 2a.

### 3.3 Surface Roughness and Hardness of Electrodeposited Ni–Se Coatings

**Figure 4:**
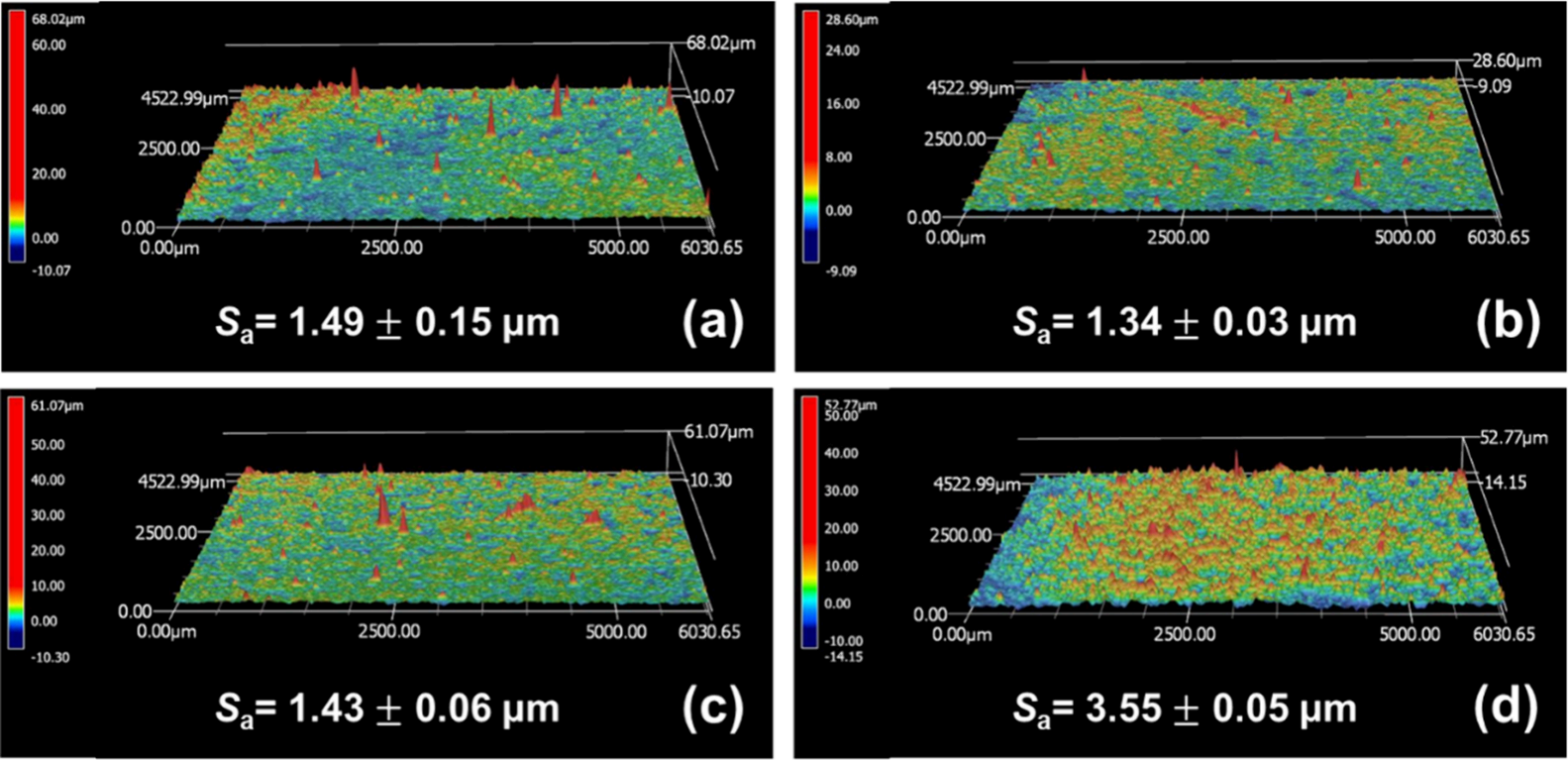
3D topographic images and the calculated surface roughness (*S*_a_) of (a) NS-40, (b) NS-50, (c) NS-60, and (d) NS-70 samples.

The surface 3D topographic images and calculated arithmetic mean heights (*S*_a_) of the electrodeposited Ni–Se coatings are shown in Figure 4. It can be observed that the NS-50 sample has a relatively smoother surface (Figure 4b), while the NS-70 sample has the roughest surface. The calculated *S*_a_ values, ranked from highest to lowest, are NS-70 > NS-40 > NS-60 > NS-50. The extreme *S*_a_ value for the NS-70 sample is likely due to the uneven reduction reaction of a large amount of Se on the cathode surface at higher bath temperatures during coating formation and the nonuniform redeposition of Se species under the electric field. The hardness of the Ni–Se coatings prepared at different bath temperatures are shown in Table 1. There is no specific trend correlating with increasing temperature. The hardness values, ranked from highest to lowest, are NS-70 > NS-50 > NS-40 > NS-60, with values ranging from approximately 430 to 543 HV. These values are slightly higher than those of pure Ni coatings prepared by conventional electrodeposition methods (around 350 HV).^23^ A comparison of the microhardness of various selenides is shown in Table S1 of the Supporting Information,^36−39^ which indicates that the Ni–Se intermetallic compounds exhibit relatively higher hardness due to the intrinsic better mechanical properties of metallic Ni.

**Table 1:** Surface Microhardness of Ni–Se Coatings Prepared at Different Process Temperatures

	NS-40	NS-50	NS-60	NS-70
hardness (HV ± σ)	469.45 ± 56.01	477.53 ± 49.75	430.43 ± 44.46	542.12 ± 114.24

### 3.4 Corrosion Behavior of Electrodeposited Ni–Se Coatings

**Figure 5:**
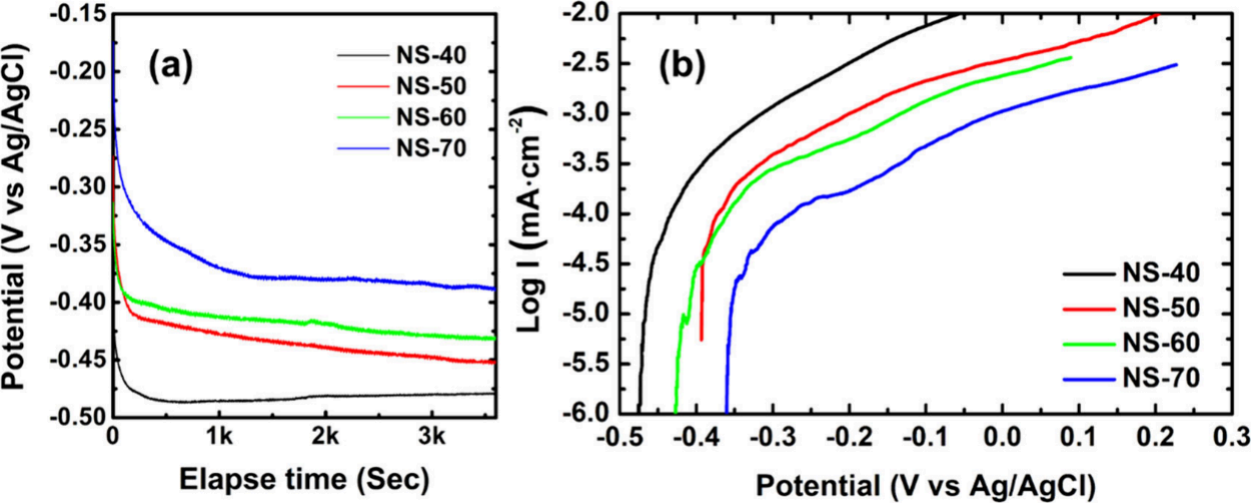
Comparison of (a) OCP and (b) anodic polarization curves for Ni–Se coatings prepared at different process temperatures.

The OCP and PDP tests were conducted to evaluate the electrochemical corrosion properties of electrodeposited Ni–Se coatings. The PDP measurements were conducted in two stages. In the first stage, the applied potential window was ±300 mV around the OCP equilibrium potential after 1 h of immersion in 3.5 wt % NaCl electrolyte. In the second stage, the range was expanded to ±600 mV around the equilibrium potential. The OCP curve variations and PDP measurement results for the first stage are shown in Figure 5. According to Figure 5a, the OCP values of all Ni–Se coatings rapidly decreased within the first 500 s of testing and then stabilized. The observed potential drop during the early stages of the OCP measurement is likely related to the reaction involving the dissolution of selenium from the Ni_*x*_Se_*y*_ coating during the immersion in water-based electrolyte. This can be indirectly supported by the stable OCP potentials typically exhibited by Ni alloy coatings in saline environments, which generally range from −0.4 to −0.7 V versus Ag/AgCl|sat. KCl.^23^ The dissolution of some Se ions from the Ni_*x*_Se_*y*_ coating results in the sample demonstrating a potential that increasingly approaches the stable OCP of pure Ni or Ni alloys as the immersion time increases. During the 1 h measurement, the NS-70 sample had the highest OCP equilibrium potential, followed by NS-60, NS-50, and NS-40. This indicates a positive correlation between the OCP equilibrium potential and the proportion of NiSe_2_ phase in the coating (as shown in panels b–e of Figure 2). In other words, the lower the Ni_0.85_Se/NiSe_2_ ratio in the coating, the higher the equilibrium potential. Generally, the OCP equilibrium potential of an electrode material after long-term immersion in the electrolyte is close to the corrosion potential obtained from PDP analysis.^40^ Therefore, it can be inferred that NiSe_2_ crystals may have relatively better thermodynamic stability compared to Ni_0.85_Se crystals.

The comparison of anodic polarization curves for Ni–Se coatings prepared at different process temperatures is shown in Figure 5b, and the calculated corrosion potential (*E*_corr_) and corrosion current density (*I*_corr_) are listed in Table 2. Among all samples, the NS-70 sample exhibited the highest corrosion potential (*E*_corr_ = −0.362 mV versus Ag/AgCl|sat. KCl), while the NS-40 sample had the lowest corrosion potential (*E*_corr_ = −0.476 mV versus Ag/AgCl|sat. KCl), which is consistent with the previous OCP results. Additionally, the corrosion current density varied significantly with the process temperature, which ranged from 16.87 to 105.07 μA cm^–2^. The NS-60 sample demonstrated the best corrosion resistance (*I*_corr_ = 16.87 μA cm^–2^), whereas the NS-50 sample showed the poorest anticorrosion property (*I*_corr_ = 105.07 μA cm^–2^). The variations in corrosion current density did not exhibit a specific trend related to the proportion of NiSe_2_ phase in the coatings or the surface roughness of the samples. However, when comparing the samples by grouping them into (1) Ni_0.85_Se (NS-40) phase and (2) Ni_0.85_Se/NiSe_2_ mixed phase (NS-50, NS-60, and NS-70), a positive correlation can be observed between the changes in corrosion current density and the Se content in the coatings, as shown in Figure 1k. Specifically, for group 2 samples, higher Se content in the coating corresponded to lower corrosion current density. The Se content in the NS-60 sample (50.8 atomic %) was slightly higher than in the NS-70 sample (50.5 atomic %), which resulted in similar corrosion current densities. In contrast, the Se content in the NS-50 sample was about 3% lower (46.9 atomic %), approximately ten times the difference in Se content between the NS-60 and NS-70 samples, which thus led to its relatively poorer corrosion resistance (*I*_corr_ = 105.07 μA cm^–2^). On the other hand, the NS-40 sample (group 1), primarily composed of the Ni_0.85_Se phase, exhibited higher corrosion current density compared to the NS-60 and NS-70 samples. This suggests that the NiSe_2_ phase has superior corrosion resistance compared to the Ni_0.85_Se phase. The better corrosion resistance of the NS-40 sample relative to the NS-50 sample may be due to the direct reduction of pure Ni in the coating. Electrodeposited Ni alloys generally possess excellent corrosion resistance and are often used as industrial anticorrosion coatings.^41^ According to the deposition mechanism shown in Figure 2f, during electrodeposition in a citric acid-ammonia electrolyte system, the formation of the coating inevitably involves the generation of pure metallic Ni. Experimental results confirmed that electrodeposition at lower temperatures resulted in higher Ni content in the coatings compared to those prepared at higher bath temperatures (as shown in Figure 1k). The NS-40 sample, with higher Ni content, as shown in Figure S4 of the Supporting Information, had some pure metallic Ni exposed on the surface in addition to Ni–Se crystals. Therefore, it is inferred that the corrosion current density measured in the PDP test for this sample may be a comprehensive contribution of metallic Ni and Ni–Se crystals, which resulted in a lower *I*_corr_ value compared to the NS-50 sample.

**Table 2:** Comparison of Corrosion Potentials and Calculated Corrosion Current Densities of Ni–Se Coatings Prepared at Different Process Temperatures

	NS-40	NS-50	NS-60	NS-70
–*E*_corr_ (V)	0.476	0.394	0.428	0.362
*I*_corr_ (μA cm^–2^)	42.35	105.07	16.87	27.74

**Figure 6:**
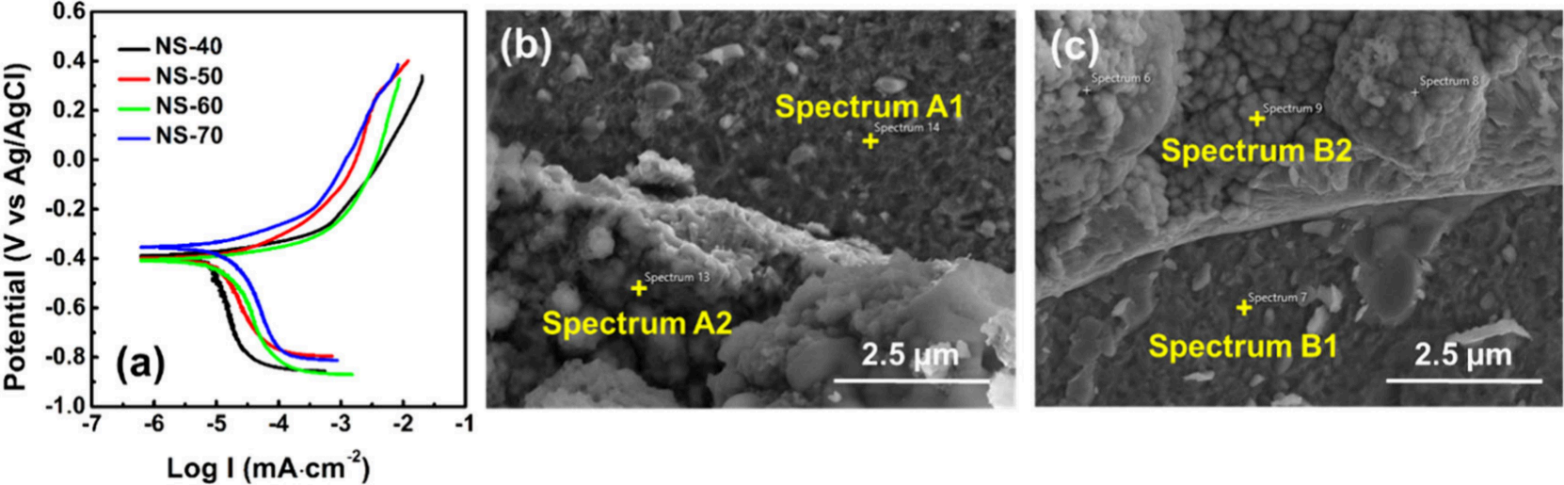
(a) Comparison of PDP curves (test range: OCP ± 600 mV) for Ni–Se coatings fabricated with different process temperatures. Panels b and c show the FE-SEM micrographs of NS-50 and NS-70 samples after PDP test (test range: OCP ± 600 mV), respectively.

The results of the second stage PDP test (test range: OCP ± 600 mV) are shown in Figure 6a. After the second stage PDP test, significant dissolution was observed on the sample surfaces, which occurred mainly in the voltage range of −0.9 to −0.6 V versus Ag/AgCl|sat. KCl. The polarization curves indicate that the corrosion potential range obtained from both stages of testing is similar (approximately 0.35–0.50 V versus Ag/AgCl|sat. KCl). A nearly horizontal plateau was observed at an overpotential near −0.9 V versus Ag/AgCl|sat. KCl in the cathodic polarization region, which suggests strong reduction reactions occurring at this potential, which is likely associated with the severe dissolution reactions observed during the test. Table S2 of the Supporting Information compares the measured weight and weight loss of the samples before and after the second stage PDP test. It shows that the weight loss of the samples decreases with increasing coating process temperature. The NS-40 sample exhibited the greatest weight loss (*W*_loss_ = 0.0026 g), approximately three times that of the NS-70 sample (*W*_loss_ = 0.0009 g), which indicates that within the test range of OCP ± 600 mV, the corrosion resistance of the Ni–Se coating is still influenced by the Ni_0.85_Se/NiSe_2_ ratio in the coating. The higher the proportion of NiSe_2_, the better the corrosion resistance of the coating, which is similar to the trend observed in the first stage results (test range: OCP ± 300 mV). However, due to the potential for localized coating peeling caused by nonuniform surface dissolution and the possible formation of surface oxides and hydroxides, it is not reasonable to directly quantify the corrosion reaction rate of the coating based solely on weight loss. The data presented here should only be used as a reference for evaluating the corrosion behavior of the coating under specific operating potentials in the saline system.

The surface microstructure of the NS-50 and NS-70 samples after the PDP test is shown in panels b and c of Figure 6, respectively. FE-SEM images reveal partial layer peeling, and the originally sharp-edged grain agglomerates have become rounded and blurred due to corrosion. To further confirm the chemical composition in different areas, EDS point analyses were conducted on the peeled areas (spectra A1 and B1) and the rest coating sides (spectra A2 and B2), as shown in Table 3. In the peeled areas of both samples, the main elements detected were Fe, and the NS-50 sample also showed a small amount of Cr signal. This suggests that these areas are associated with the SUS420 stainless-steel substrate, and the detected O signal is likely related to surface oxidation caused by immersion in the saline electrolyte during the test. Additionally, Na, Cl, and trace amounts of Ni and Se were detected in spectra A1 and B1, which represent residual salts from the electrolyte and characteristics of the remaining Ni–Se coating. Conversely, the chemical composition on the rest coating sides of both samples differed significantly from the pre-PDP test results. In addition to signals from residual salts (Na and Cl), Fe was detected, which indicates thinning of the coating due to corrosion during the electrochemical tests. Before the PDP test, EDS signals only showed Ni and Se, whereas after saline immersion, significant O signals appeared at the coating sides. This phenomenon is related to the oxidation and/or hydroxylation of Ni and Se on the sample surfaces. In comparison of the Ni/Se ratios before and after the PDP test for the NS-50 and NS-70 samples, both samples had pretest ratios close to 1 (1.13 for NS-50 and 0.98 for NS-70). After testing, the ratios changed to approximately 1.78 and 0.74, respectively. This indicates a more severe loss of Se in the NS-50 sample during corrosion, likely due to dissolution reactions. In contrast, the Se content in the NS-70 sample remained higher than Ni, which suggests milder corrosion reactions. Because the corrosion of metallic Ni in saline systems is minimal (typically limited to minor pitting corrosion),^42^ it is inferred that the change in the Ni/Se ratio is related to the oxidation and/or hydroxylation of surface Se elements, where Se reacts with the electrolyte, which covers the original Ni–Se coating and results in a decreased Ni/Se ratio. Notably, due to the dissolution and peeling of the coating after the second stage PDP test, parts of the substrate are exposed to the electrolyte. The measured polarization curves (especially in the anodic polarization region) therefore include the electrochemical characteristics of the substrate and do not purely represent the properties of the Ni–Se compounds. Consequently, Tafel extrapolation was not used to calculate corrosion current density here to evaluate the overall corrosion rate of the samples.

**Table 3:** Comparison of Surface Elemental Composition for NS-50 and NS-70 Samples after PDP Test at Different Regions (PDP Test Range: OCP ± 600 mV)

sample	element (atomic %)	Ni	Se	Fe	Cr	O	Cl	Na
NS-50	spectrum A1	0.3	1.6	72.8	8.5	13.7	0.0	3.1
	spectrum A2	22.3	12.5	11.5	3.7	45.5	2.6	2.0
NS-70	spectrum B1	0.0	1.3	94.3	0.0	3.9	0.5	0.0
	spectrum B2	32.8	44.2	4.7	0.0	17.7	0.6	0.1

**Figure 7:**
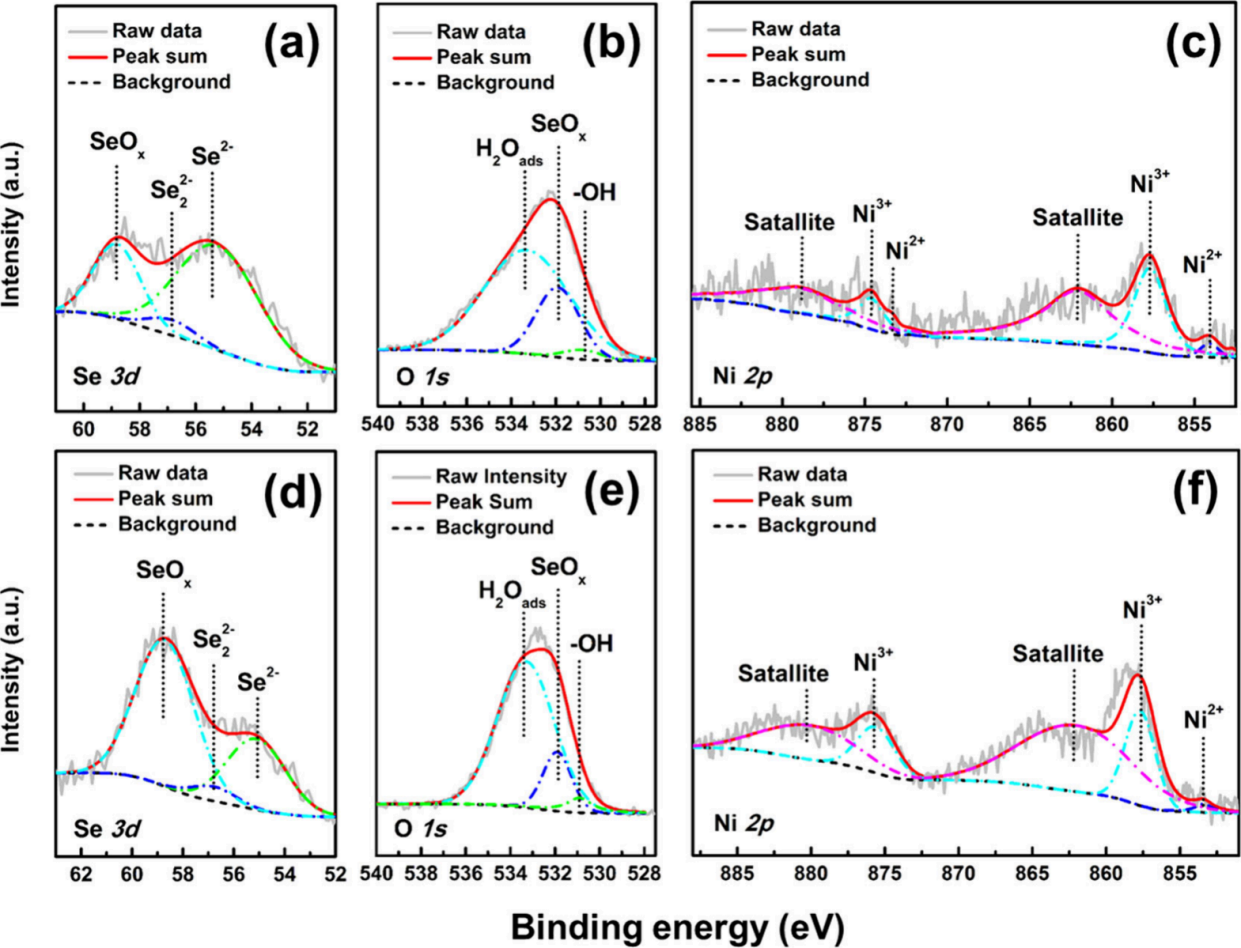
Panels a–c illustrate XPS spectra in Se 3d, O 1s, and Ni 2p orbits of NS-50 sample after PDP test, respectively. Panels d–f are core-level Se 3d, O 1s, and Ni 2p regions of NS-70 sample after PDP test, respectively (PDP test range: OCP ± 600 mV).

To further understand the changes in valence states on the sample surface after corrosion testing, XPS analysis was conducted again on the NS-50 and NS-70 samples following the PDP test (test range: OCP ± 600 mV), and the results are shown in Figure 7. Panels a and d of Figure 7 illustrate the peak fitting results of the Se 3d orbital for the NS-50 and NS-70 samples, respectively, which display distinct characteristics. The peak area ratios in the Se 3d orbital before and after the PDP test for both samples are compared in Table S3 of the Supporting Information. It is observed that the core-level Se 3d region for both samples after PDP test still consists of Se^2–^, Se_2_^2–^, and SeO_*x*_ features. The XPS results of the NS-50 sample before and after corrosion exhibit a noticeable weakening of the peaks corresponding to Se_2_^2–^ and SeO_*x*_ species. On the basis of the previous analyses, it can be inferred that a substantial amount of Se was lost during corrosion, which corroborates the inference drawn from Tables 2 and 3 and Table S2 of the Supporting Information. On the other hand, comparing the XPS results of NS-70 sample before and after the PDP test, a similar weakening of the Se_2_^2–^ peak can be observed, but the proportion of the SeO_*x*_ peak significantly increases from 14.9 to 63.9%, and the combined proportion of Se_2_^2–^ and SeO_*x*_ increases from 57.4 to 68.7%. On the basis of the inference obtained from the EDS analysis shown in Table 3, the reduction in the Se_2_^2–^ peak intensity can be attributed to the dissolution reaction during corrosion. It is likely that some Se_2_^2–^ species converted to SeO_*x*_ during the reaction and resulted in an intensified surface SeO_*x*_ signal in Se 3d orbital after the PDP test. This means the Se that did not dissolve may have oxidized and remained on the coating surface probably due to the sluggish dissolution reaction. The as-formed surface SeO_*x*_ can act as a protective layer for the inner Ni–Se crystals. Therefore, a better corrosion resistance (as shown in Table 2) and a less weight loss can be observed in the NS-70 sample compared to the NS-50 sample (as shown in Table S2 of the Supporting Information). Panels b and e of Figure 7 show the O 1s orbital characteristics of the NS-50 and NS-70 samples after corrosion, which reveal significant differences compared to before corrosion. Both samples show a great enhancement of the peaks corresponding to physically/chemically adsorbed water molecules. This phenomenon is particularly evident in the NS-70 sample, which has a higher Se content, indicating a high affinity for water molecules by the Se and Ni hydroxyl groups formed after corrosion. In the Ni 2p orbital region (panels c and f of Figure 7), the spectra are mainly dominated by Ni^3+^, and include features corresponding to Ni^2+^ and shakeup satellites. Therefore, it can be inferred that during the electrochemical reaction process, nickel primarily undergoes oxidation and hydroxidation, while the corrosion behavior of the Ni–Se coating is predominantly driven by reactions involving the selenium.

### 3.5 Corrosion Mechanism of Electrodeposited Ni–Se Coatings

**Figure 8:**
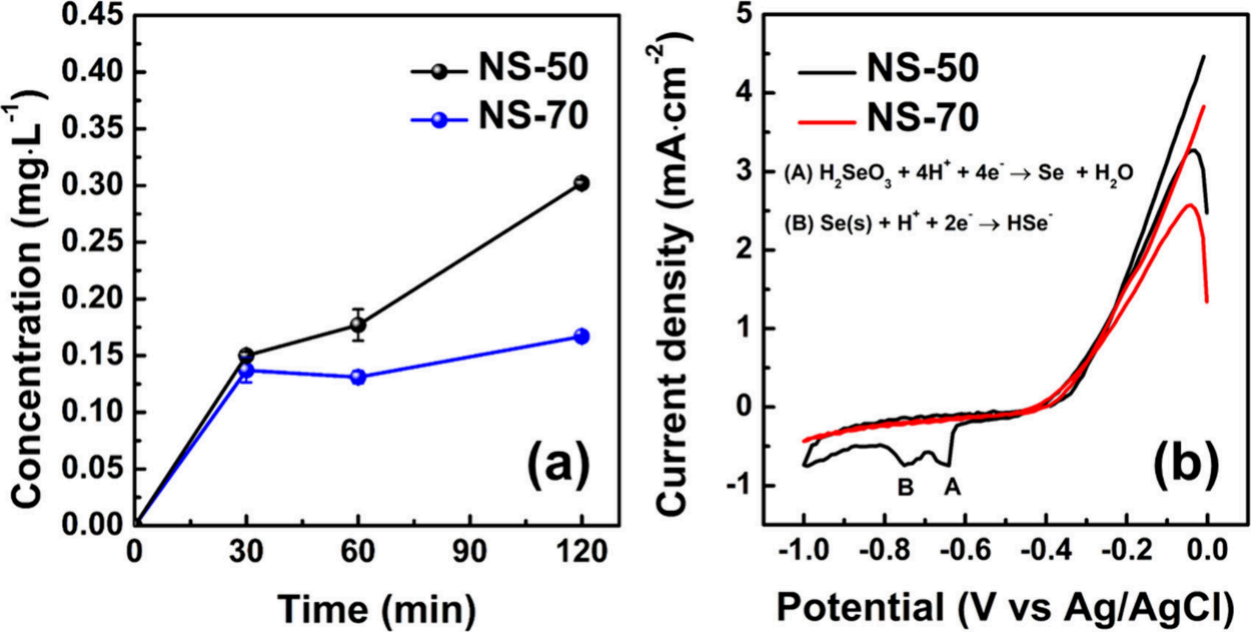
(a) Se concentration variation in DI water for NS-50 and NS-70 samples within 2-h immersion. (b) CV plot showing the CV curves of NS-50 and NS-70 samples from 0.0 to −1.0 V versus Ag/AgCl|sat. KCl in 3.5 wt % NaCl solution.

From the experimental results presented in the previous sections, it is evident that the electrodeposited Ni–Se coating undergoes corrosion due to the interaction between the Se and the water-based electrolyte under different potential loads. This phenomenon is particularly severe in samples prepared at a process temperature of 50 °C. An intriguing question arises: “Is this corrosion phenomenon related to the applied voltage load, or is it purely driven by the reaction between the Se element and water molecules?” To clarify this, the NS-50 and NS-70 samples were immersed in DI water without applying potential, and ICP–OES analysis was then conducted on the electrolyte at different immersion times to evaluate the chemical composition of the dissolved species and their concentrations. As shown in Figure 8a, the results indicate that after a certain period of immersion in DI water, both NS-50 and NS-70 samples exhibited Se element dissolution after 30 min immersion, and the concentration of Se in DI water increased with prolonged immersion time. The Se concentration curves for both samples in the immersion water reveal that the initial Se dissolution reaction was the most intense within the entire experimental period, with the NS-50 sample showing a higher Se dissolution amount thereafter. After 2 h of immersion, the Se dissolution amounts for the NS-50 and NS-70 samples were 0.302 and 0.167 mg L^–1^, respectively, with the former being nearly twice that of the latter. This indicates poorer corrosion resistance of the NS-50 sample when immersed in DI water without an applied electric field. It is hypothesized that this natural corrosion phenomenon is related to the formation of Se oxides and hydroxides (e.g., SeO_2_ and H_2_SeO_3_) on the surface of the Ni–Se coating, which are soluble in water under ambient conditions. Consequently, the dissolution amount increases over time during the immersion process. However, when the sample contains certain amount of Se (e.g., NS-70), due to the formation of a large amount of SeO_*x*_ and its sluggish dissolution reaction, some SeO_*x*_ would still remain on the coating surface and act as a protective layer for the underlying Ni–Se crystals. Therefore, the NS-50 sample exhibited a much higher corrosion rate than the NS-70 sample, even at relatively lower reaction potentials, as shown in Figure 5b.

To determine whether the dissolution reaction of Se in Ni–Se coatings is influenced and intensified by an electric field, this study conducted CV tests on the NS-50 and NS-70 samples. The comparison of the test curves is shown in Figure 8b. The NS-50 and NS-70 samples both exhibit a distinct reduction peak around −0.1 V at the initial scanning stage. This reduction peak is regarded to be related to the transition between Se-based species, represented by the reaction HSeO_3_^–^ + 6H^+^ + 6e^–^ = HSe^–^ + 3H_2_O.^43^ Apart from this peak, the NS-50 and NS-70 samples display distinctly different current response characteristics within the measurement range of 0.0 to −1.0 V versus Ag/AgCl|sat. KCl. The NS-50 sample exhibits two prominent reduction peaks at overpotentials of −0.64 and −0.74 V versus Ag/AgCl|sat. KCl, corresponding to the redox reactions (A) H_2_SeO_3_ + 4H^+^ + 4e^–^ → Se + H_2_O and (B) Se(s) + H^+^ + 2e^–^ → HSe^–^.^44,45^ In contrast, the NS-70 sample shows no significant redox peaks in the entire scanning range, which indicates that the applied electric field does not significantly enhance the dissolution of Se species in the NS-70 sample. According to the XRD results in panels a–e of Figure 2, the NS-50 and NS-70 samples are primarily dominated by Ni_0.85_Se and NiSe_2_ phases, respectively. In combination with the ICP–OES results in Figure 8a, it can be inferred that these two crystal phases exhibit different corrosion characteristics under negative potential load (i.e., 0.0 to −1.0 V versus Ag/AgCl|sat. KCl). The NS-50 sample (dominated by the Ni_0.85_Se phase) undergoes both natural dissolution of Se species in water and intensified corrosion under specific potential loads, which therefore results in poorer corrosion resistance compared to the NS-70 sample (dominated by the NiSe_2_ phase).

**Figure 9:**
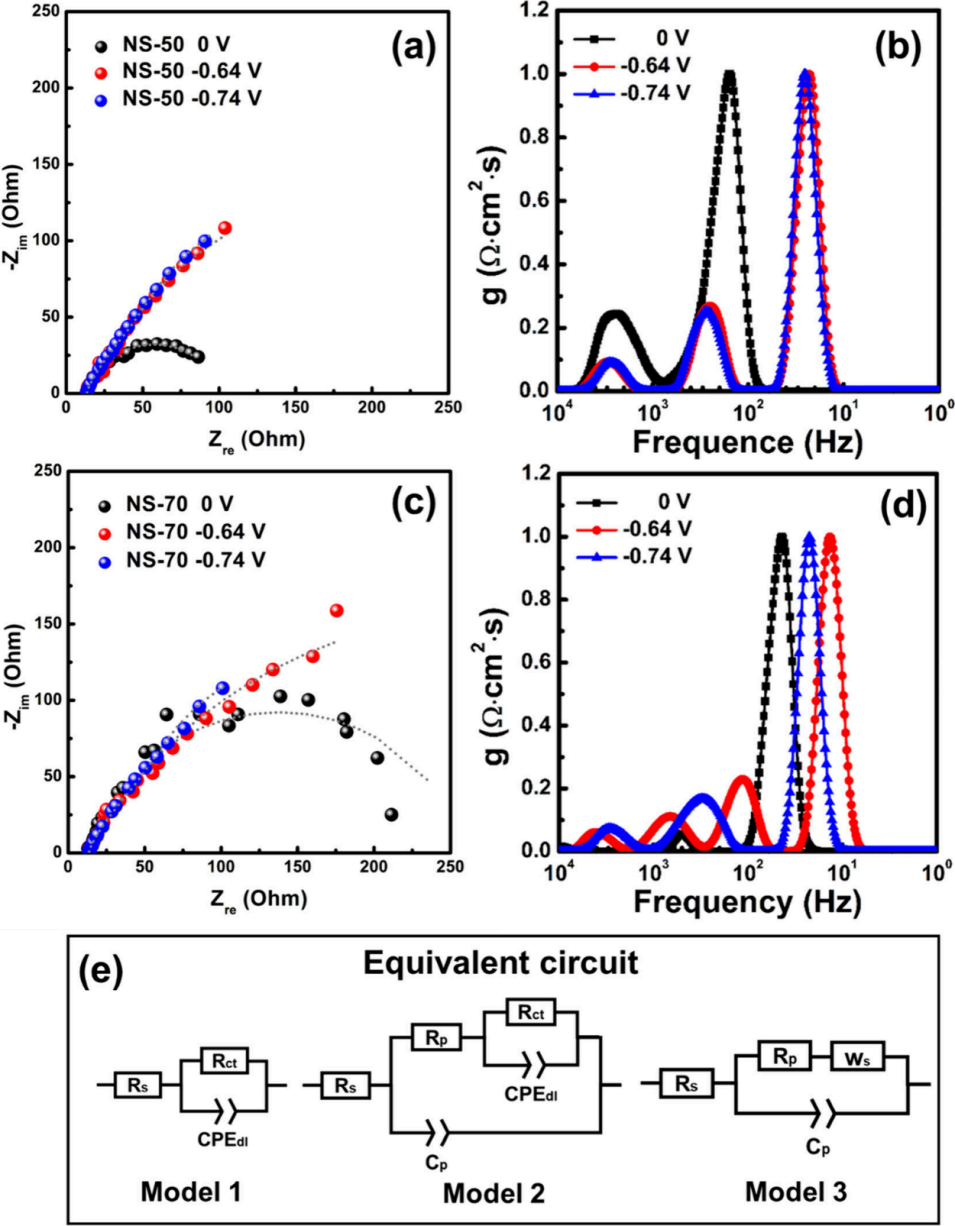
(a) EIS study for NS-50 sample at 0, −0.64, and −0.74 V in 3.5 wt % NaCl solution. (b) DRT curve of NS-50 sample. Panel c illustrate the Nyquist plots of NS-70 sample examined at 0, −0.64, and −0.74 V in 3.5 wt % NaCl solution. (d) DRT curve of NS-70 sample. (e) Equivalent circuits used for EIS data fitting.

From the CV analysis in Figure 8b, it is evident that the Ni–Se coating exhibits intensified corrosion reaction at specific potential loads. To understand the corrosion kinetics of the samples under individual potential loads, EIS analysis was further conducted on the NS-50 and NS-70 samples, with the results presented as Nyquist plots in Figure 9. The fitting parameters are shown in Table S4 of the Supporting Information. In these parameters, *R*_s_, *R*_p_, and *R*_ct_ represent the solution resistance (Ω), corrosion product resistance (Ω), and charge transfer resistance (Ω), respectively. *C* denotes the ideal capacitance, while *C*_p_ and *C*_dl_ represent the corrosion product capacitance (mF) and double-layer capacitance (mF), respectively. CPE is the constant phase element used to describe sluggishly electrochemical kinetics due to irregular geometric features on the electrode surface, with CPE_dl_–*T* and CPE_dl_–*P* indicating the imaginary capacitance (*Z*_CPE_ = [*T*(*j*ω)^P^]^−1^, where *P* is the power of CPE, ranging from 0 to 1) and its deviation from the ideal capacitance. ω is the angular frequency. The unit of CPE is mF s^*P*–1^. *W*_s_ is the finite-length Warburg element, which represents mass transfer phenomena at the electrode/electrolyte interface. It consists of a diffusion resistance (*W*_s_–*R*, Ω) in series with an imaginary diffusion capacitance, with *W*_s_–*T* defined as *L*^2^*D*^–1^ (s), where *L* is the diffusion layer thickness (cm) and *D* is the diffusion coefficient (cm^2^ s^–1^). *W*_s_–*P* is the power for calculating the imaginary diffusion capacitance.

Without applying an electric field, both samples exhibited uniform corrosion and nearly semicircular geometric features. Fitting the data with the *R*_s_(*R*_ct_//CPE_dl_) circuit in Figure 9e shows that the solution resistances for both samples are approximately 13 Ω. The charge transfer resistance at the electrode/electrolyte interface for the NS-50 and NS-70 samples is 88.7 Ω and 238.1 Ω, respectively, which indicates that the NS-70 sample has relatively better corrosion resistance. This finding aligns with the results in Table 2, Figure 8, and Table S2 of the Supporting Information. Using the following formula, the CPE parameters were converted into ideal capacitance values.^46^
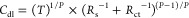
5The calculated *C*_dl_ values for the NS-50 and NS-70 samples are 1.864 and 0.306 mF, respectively. Because the *C*_dl_ value is proportional to the number of active sites on the electrode surface, it can be inferred that the natural dissolution reaction of Se in the NS-50 sample is more intense than in the NS-70 sample. This result is consistent with the trend shown in Figure 8a. Under the applied voltage of −0.64 V versus Ag/AgCl|sat. KCl, the Ni–Se electrodes exhibited impedance response that was distinctly different from the condition without applied voltage. In panels a and c of Figure 9, both NS-50 and NS-70 samples showed lines with high curvature radius. From the previous CV analysis, it is evident that at this potential, there might be a reduction reaction of selenic acid near the electrode surface (i.e., H_2_SeO_3_ + 4H^+^ + 4e^–^ → Se + H_2_O). As the exact location of the reduction reaction cannot be predicted, the *R*_s_(*C*_p_//*R*_p_(*R*_ct_//CPE_dl_)) circuit in Figure 9e was used to describe the surface layer formed by the nonuniform reduction reaction. The fitting results in Table S4 of the Supporting Information show that the solution resistance for both NS-50 and NS-70 samples is around 13 Ω. The first time constant includes a parallel circuit of *C*_p_ and *R*_p_, and another parallel circuit of *R*_ct_ and CPE_dl_. *R*_ct_ represents the interface resistance between the surface corrosion product and the original Ni–Se coating, which is much higher than the resistance of the corrosion product (*R*_p_), indicating that the corrosion product exhibits more severe corrosion behavior than the original coating. Furthermore, comparing the *R*_ct_ values of the NS-50 and NS-70 samples, the NS-70 sample still shows a relatively higher value (543.5 Ω), approximately 1.4 times that of the NS-50 sample (391.7 Ω), which indicates its superior corrosion resistance at this potential. When the applied voltage is increased to −0.74 V versus Ag/AgCl|sat. KCl, the curvature of the lines in the Nyquist plots for both NS-50 and NS-70 samples decreases compared to −0.64 V versus Ag/AgCl|sat. KCl (panels a and c of Figures 9), which indicates more linear characteristics. From the CV analysis in Figure 8b, it can be inferred that the reaction at this potential might be related to Se(s) + H^+^ + 2e^–^ → HSe^–^, which reflects the redissolution behavior of the original Se on the coating surface and the Se generated through the reduction reaction. Using the *R*_s_(*C*_p_//*R*_p_(*W*_s_)) circuit in Figure 9e for numerical fitting, it is observed that the solution resistance remains at around 13 Ω. Due to the intense reduction reaction of Se substances at this potential, the *R*_p_ value (approximately 0.8 Ω) is about 20 times smaller than at −0.64 V versus Ag/AgCl|sat. KCl. The NS-70 sample shows slightly higher diffusion impedance (*W*_s_–*R*) compared to the NS-50 sample, which suggests that the diffusion reaction of Se substances on the NS-50 sample surface is somewhat more intense at this potential. However, both samples exhibit the same *W*_s_–*T* and *W*_s_–*P* values, which implies that the extent of the diffusion reaction is similar for both samples. This indicates that at this potential, significant Se substance redissolution reactions occur regardless of whether the coating is dominated by the Ni_0.85_Se or NiSe_2_ phase.

In the past, analyses of EIS data have often relied on fitting Nyquist data to equivalent models. The advantage of this method lies mainly in its ability to describe the reactions in the electrode system during the electrochemical process in a layered manner. However, for some complex electrochemical systems, surface and near-surface reactions might occur simultaneously, which leads to ongoing debates about the applicability of the fitting circuits. To address this issue, the distribution function of relaxation times (DRT) method has been developed in recent years. The advantage of using this method is that the time-domain information can more easily distinguish the time constants of electrochemical reactions compared to fitting with imaginary circuits. DRT method can verify EIS fitting models and identify overlapping time constants. For the representative examples, it has been evidenced applicable for analyzing EIS data in lithium-ion batteries (LIBs), solid oxide fuel cells (SOFCs), and electrochemical catalysis (EC).^47−49^ However, studies related to corrosion are relatively scarce and mainly focus on magnesium alloy corrosion.^50,51^ Therefore, this study further converted the impedance spectra data of the NS-50 and NS-70 samples measured at different potentials into DRT, and the results are shown in panels b and d of Figure 9.

The DRT fitting curves for both samples without applied voltage contain two characteristic peaks. The weaker peak at around 1–10 kHz in the high-frequency region might be related to surface instability and external electromagnetic signal interference during measurement.^52^ The second peak in the mid-to-high frequency range (10–1000 Hz) corresponds to the *R*_ct_//CPE_dl_ time constant in the model 1 equiv circuit. In comparison of the characteristic peak frequencies in the mid-to-high frequency range for both samples, the NS-70 sample shows a relatively lower characteristic peak frequency, which indicates a longer transition time from transient to steady-state electrochemical reactions, suggesting better corrosion resistance. This result aligns with the *I*_corr_, Se dissolution, and *R*_ct_ data presented in Table 2, Figure 8a, and Table S4 of the Supporting Information, respectively. Under the applied voltage of −0.64 V versus Ag/AgCl|sat. KCl, the DRT fitting curves for the NS-50 and NS-70 samples, after ignoring high-frequency interference signals (1–10 kHz), show two and three characteristic peaks, respectively. The two characteristic peaks of the NS-50 sample at around 100–1000 and 10–100 Hz correspond to the *R*_p_//*C*_p_ and *R*_ct_//CPE_dl_ parallel circuits in the model 2 equiv circuit, which represent the electrochemical characteristics of the corrosion products and their interface with the original coating. The NS-70 sample contains two characteristic peaks in the mid-to-high frequency range (100–1000 Hz), which may be related to its higher Se content. In comparison of the XPS analysis results in Figures 3d and 7d, a significant decrease in Se_2_^2–^ and an increase in SeO_*x*_ species were observed in the NS-70 sample after corrosion. Thus, during the electrochemical process, the reduction reaction of H_2_SeO_3_/Se on the sample surface might be accompanied by the oxidation behavior of Se/SeO_*x*_, which increases the sample surface impedance during measurement and results in distinct DRT peak characteristics and prolonged relaxation times in the mid-to-low frequency range (around 10 Hz). When the potential load is increased to −0.74 V versus Ag/AgCl|sat. KCl for EIS measurement, the DRT fitting curves for both NS-50 and NS-70 samples show three characteristic peaks, with the frequency ranges of each peak being similar. Aside from the weak high-frequency signal (1–10 kHz), the characteristic peaks at 100–1000 and 10–100 Hz correspond to the *R*_p_//*C*_p_ parallel circuit and Warburg impedance in the model 3 equiv circuit, possibly related to the reduction of H_2_SeO_3_ and the redissolution of Se elements. It should be noted that the description of DRT fitting data in this study mainly references the previous material properties and electrochemical properties analyses. However, because the explanation of the corrosion behavior of Ni–Se intermetallic compounds and related DRT phenomena is still in the early stages, further verification is needed through supplementary analysis methods such as quartz crystal microbalance (QCM) and scanning electrochemical microscopy (SECM).

In summary, Ni–Se intermetallic compounds are highly sensitive to water-based electrolytes, and their corrosion reactions differ significantly depending upon the dominant crystalline phase and chemical composition. Therefore, despite their great potential in applications such as electrochemical catalysis and supercapacitors, corrosion prevention measures are indispensable. Coating an additional surface protective layer and introducing more Se into the Ni–Se coatings have been proven feasible,^53,54^ and further research into their fundamental properties and performance enhancement is warranted.

## 4 Conclusion

In this study, Ni–Se intermetallic compound coatings were prepared using electrodeposition, and the crystalline phase of coating was controlled by adjusting the process temperature. The results show that coatings prepared at 40–50 °C exhibit the characteristics of Ni_0.85_Se phase. Increasing the process temperature promotes the deposition of Se elements and the formation of the NiSe_2_ phase but also leads to an increase in surface roughness. Both Ni_0.85_Se and NiSe_2_ phase-dominated samples exhibited Se dissolution in pure water, with the amount of Se dissolved decreasing as the process temperature increased, which indicates that Ni_0.85_Se phase-dominated samples exhibited relatively stronger natural corrosion reactions. Polarization curves measured in NaCl electrolyte within the test range of OCP ± 300 mV indicate that NiSe_2_ has better corrosion resistance compared to Ni_0.85_Se. Expanding the test range to OCP ± 600 mV shows that Ni–Se samples experience intense corrosion reactions in the low potential region. According to the CV test results, Ni_0.85_Se phase-dominated samples exhibit significantly enhanced Se transformation/dissolution reactions at voltages of −0.64 and −0.74 V versus Ag/AgCl|sat. KCl, a phenomenon less apparent in NiSe_2_ phase-dominated samples. Further EIS analysis to evaluate the electrochemical kinetics of the samples under different potential loads confirms that NiSe_2_ phase-dominated samples indeed have better corrosion resistance. However, both Ni_0.85_Se and NiSe_2_ phase-dominated samples experience intensified corrosion under potential loads of −0.64 and −0.74 V versus Ag/AgCl|sat. KCl, with Ni_0.85_Se phase-dominated samples being particularly severe. It is speculated that the corrosion resistance is highly related to the Se content in the coating. The higher proportion of Se content in the NiSe_2_ phase undergoes a two-stage transformation/dissolution process during electrochemical reactions, which mitigated the loss of Se and thereby exhibited relatively better corrosion resistance.

## Supporting Information Available

Cross-sectional FE-SEM–EDS mapping (Figure S1), cross-sectional FE-SEM–EDS line scan (Figure S2), XPS survey scan spectra (Figure S3), FE-SEM micrograph and EDS mapping (Figure S4), microhardness of coatings (Table S1), weight loss of coatings after PDP test (Table S2), XPS analysis in the Se 3d orbital (Table S3), and EIS fitting parameters (Table S4) (PDF)
