# Supplementary material for: Unveiling the Corrosion Mechanism of the NixSey Intermetallic Compound in Water-Based Solution
Source: ACS Appl Mater Interfaces. 2024 Nov 29;16(49):68247–61. doi: 10.1021/acsami.4c13794 (PMC11647754; doi:10.1021/acsami.4c13794)
Supplement: Supplementary file 1 — am4c13794_si_001.pdf [file am4c13794_si_001.pdf]

Supporting Information

Unveiling the Corrosion Mechanism of Ni_xSe_y Intermetallic Compound in Water-Based Solution

Jui-Teng Liang[a], *Hwai-En Lin*[a,b,c*]

[a]Institute of Mechatronic Engineering, National Taipei University of Technology, Taipei, 10608, Taiwan

[b]Graduate Institute of Manufacturing Technology, National Taipei University of Technology, Taipei, 10608, Taiwan

[c]Department of Mechanical Engineering, National Taipei University of Technology, Taipei, 10608, Taiwan

KEYWORDS NiSe, $NiSe_2$, electrodeposition, intermetallic compound, corrosion

Corresponding Author

*Hwai-En Lin – Department of Mechanical Engineering, National Taipei University of Technology, Taipei 10608, Republic of China (R.O.C.); DOI: https://orcid.org/0000-0001-5570-6738; Email: linhe@ntut.edu.tw

<u>Table of content</u>

Figure S1. Cross-sectional FE-SEM-EDS mapping of Ni-Se coatings.

Figure S2. Cross-sectional FE-SEM-EDS line scan of Ni-Se coatings.

Figure S3. XPS survey scan spectra of Ni-Se coatings.

Figure S4. FE-SEM micrograph and EDS mapping of NS-40 sample.

Table S1. Microhardness of Ni-Se coatings.

Table S2. Weight loss of Ni-Se coatings after PDP test (PDP test range: OCP $\pm$ 600 mV).

Table S3. XPS study in Se *3d* orbital for Ni-Se coatings after PDP test (PDP test range: OCP $\pm$ 600 mV).

Table S4 EIS fitting parameters for Ni-Se coatings at 0V, -0.64V, and -0.74V vs Ag/AgCl | sat. KCl in 3.5 wt.% NaCl solution.

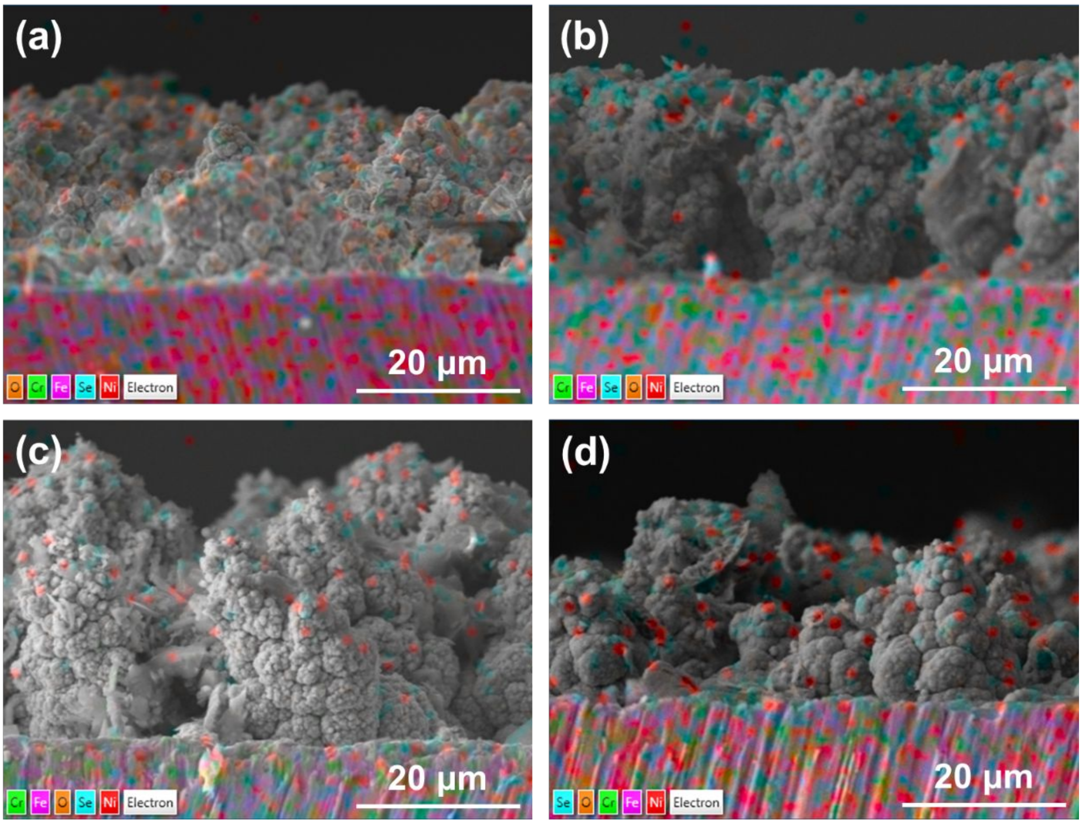

Figure S1. Cross-sectional FE-SEM-EDS mapping of (a) NS-40, (b) NS-50, (c) NS-60, (d) NS-70 samples.

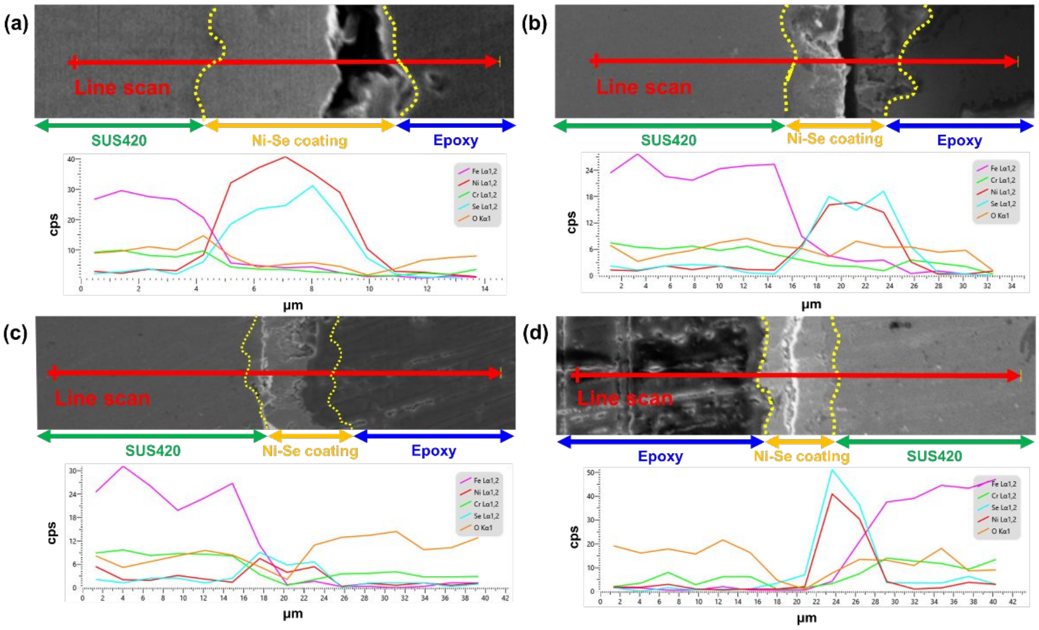

Figure S2. Cross-sectional FE-SEM-EDS line scan along with the red line of (a) NS-40, (b) NS-50, (c) NS-60, (d) NS-70 samples.

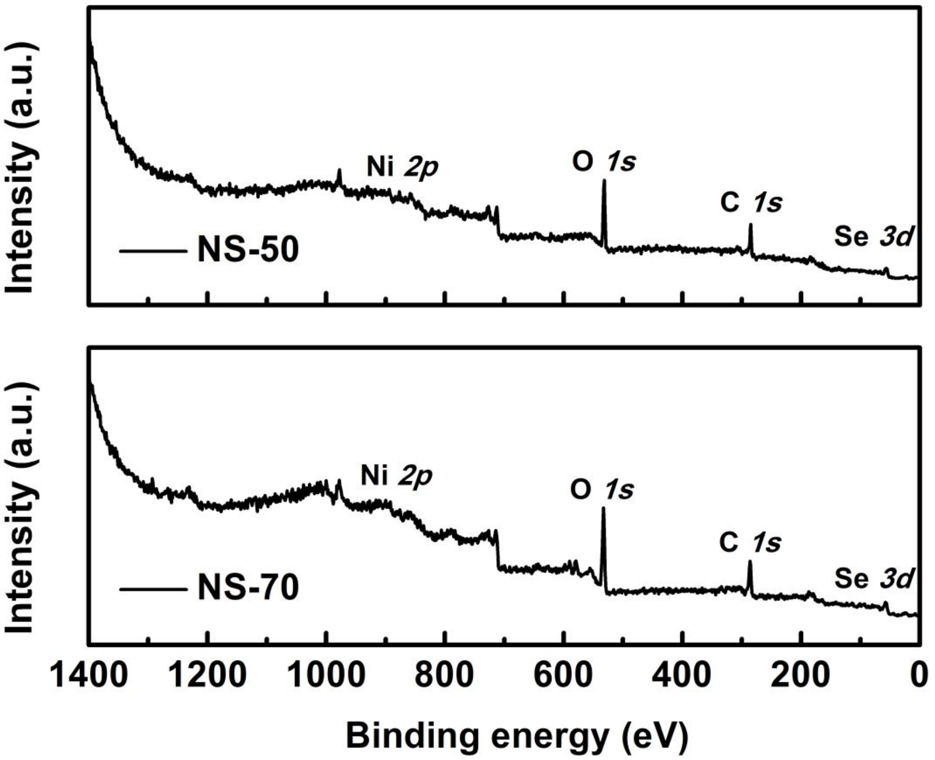

Figure S3. Comparison of XPS survey scan spectra for NS-50 and NS-70 samples.

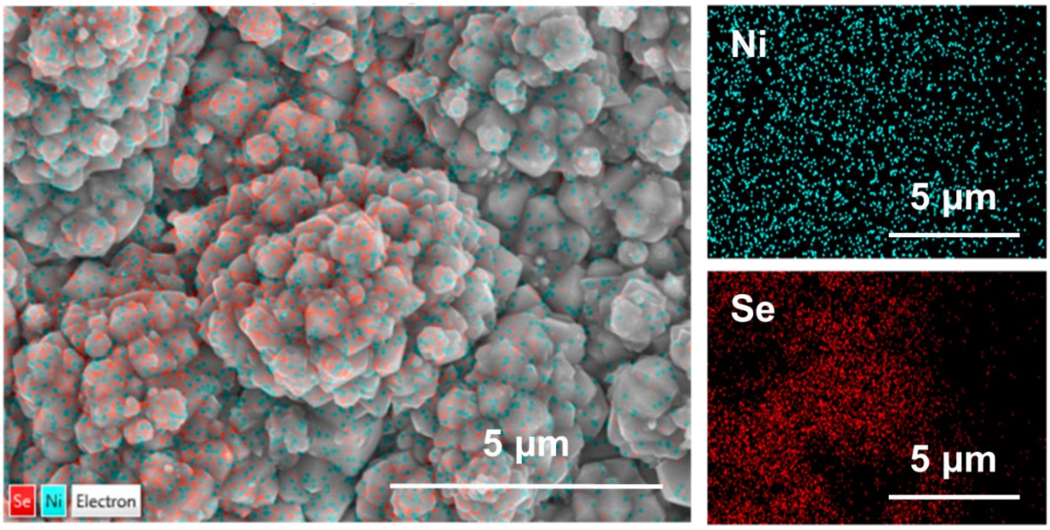

Figure S4. FE-SEM micrograph and EDS mapping of NS-40 sample.

Table S1. Comparison of microhardness obtained from Vickers hardness test for different selenides at room temperature.

Material	Hardness (GPa)	Reference
SnSe	0.27 ± 0.05	[36]
AsSe	1.04 ± 0.03	[37]
GaSe	0.25	[38]
$Cu_{2-x}Se$	1.27~2.94	[39]
NS-70	5.32 ± 1.12	-

Table S2. Weight loss of Ni-Se coatings fabricated at different process temperatures (PDP test range: OCP ± 600 mV).

Sample	Weight of sample		Weight loss (g)
	Before PDP test	After PDP test	(W_{loss})
**NS-40**	0.7107	0.7081	0.0026
**NS-50**	0.8010	0.7995	0.0015
**NS-60**	0.7710	0.7697	0.0013
**NS-70**	0.6717	0.6708	0.0009

Table S3. Comparison of calculated peak intensity ratio of Se^{2-}, Se_2^{2-}, and SeO_x species in Se *3d* orbital for NS-50 and NS-70 samples (PDP test range: OCP $\pm$ 600 mV).

Sample		Se^{2-}	Se_2^{2-}	SeO_x
NS-50	Before PDP test	39.9%	25.6%	34.5%
	After PDP test	66.9%	5.7%	27.4%
NS-70	Before PDP test	42.5%	42.5%	14.9%
	After PDP test	31.3%	4.8%	63.9%

Table S4 EIS fitting parameters for NS-50 and NS-70 samples at 0V, -0.64V, and -0.74V vs Ag/AgCl｜sat. KCl in 3.5 wt.% NaCl solution. Note: Exposure area of the working electrode was set as 1 cm^2.

		R_s (Ω)	R_{ct} (Ω)	CPE_{dl}-T ($mF \cdot s^{P-1}$)	CPE_{dl}-P	Chi-square
0V	NS-50	13.2	88.7	1.01	0.80	1.26×10^{-3}
	NS-70	13.4	238.1	0.24	0.82	4.93×10^{-3}

		R_s (Ω)	R_p (Ω)	C_p (mF)	R_{ct} (Ω)	CPE_{dl}-T ($mF \cdot s^{P-1}$)	CPE_{dl}-P	Chi-square
-0.64V	NS-50	13.6	16.5	0.23	391.7	1.42	0.71	3.19×10^{-3}
	NS-70	12.6	1.6	0.04	543.5	0.99	0.65	2.41×10^{-3}

		R_s (Ω)	R_p (Ω)	C_p (mF)	W_s-R (Ω)	W_s-T (s)	W_s-P	Chi-square
-0.74V	NS-50	13.5	0.80	0.21	262.1	0.58	0.52	1.33×10^{-3}
	NS-70	13.6	0.75	0.17	277.4	0.58	0.52	3.28×10^{-3}
